# Cell Sorting and Noise-Induced Cell Plasticity Coordinate to Sharpen Boundaries between Gene Expression Domains

**DOI:** 10.1371/journal.pcbi.1005307

**Published:** 2017-01-30

**Authors:** Qixuan Wang, William R. Holmes, Julian Sosnik, Thomas Schilling, Qing Nie

**Affiliations:** 1 Center for Complex Biological Systems, University of California Irvine, Irvine, CA, United States of America; 2 Department of Mathematics, University of California Irvine, Irvine, CA, United States of America; 3 Department of Physics and Astronomy, Vanderbilt University, Nashville, TN, United States of America; 4 Department of Developmental and Cell Biology, University of California Irvine, Irvine, CA, United States of America; EMBL-Heidelberg, GERMANY

## Abstract

A fundamental question in biology is how sharp boundaries of gene expression form precisely in spite of biological variation/noise. Numerous mechanisms position gene expression domains across fields of cells (e.g. morphogens), but how these domains are refined remains unclear. In some cases, domain boundaries sharpen through differential adhesion-mediated cell sorting. However, boundaries can also sharpen through cellular plasticity, with cell fate changes driven by up- or down-regulation of gene expression. In this context, we have argued that noise in gene expression can help cells transition to the correct fate. Here we investigate the efficacy of cell sorting, gene expression plasticity, and their combination in boundary sharpening using multi-scale, stochastic models. We focus on the formation of hindbrain segments (rhombomeres) in the developing zebrafish as an example, but the mechanisms investigated apply broadly to many tissues. Our results indicate that neither sorting nor plasticity is sufficient on its own to sharpen transition regions between different rhombomeres. Rather the two have complementary strengths and weaknesses, which synergize when combined to sharpen gene expression boundaries.

## Author Summary

In many developing systems, chemical gradients control the formation of segmental domains of gene expression, specifying distinct domains that go on to form different tissues and structures, in a concentration-dependent manner. These gradients are noisy however, raising the question of how sharply delineated boundaries between distinct segments form. It is crucial that developing systems be able to cope with stochasticity and generate well-defined boundaries between different segmented domains. Previous work suggests that cell sorting and cellular plasticity help sharpen boundaries between segments. However, it remains unclear how effective each of these mechanisms is and what their role in sharpening may be. Motivated by recent experimental observations, we construct a hybrid stochastic model to investigate these questions. We find that neither mechanism is sufficient on its own to sharpen boundaries between different segments. Rather, results indicate each has its own strengths and weaknesses, and that they work together synergistically to promote the development of precise, well defined segment boundaries. Formation of segmented rhombomeres in the zebrafish hindbrain, which later form different components of the central nervous system, is a motivating case for this study.

## Introduction

The specification of segmental domains of gene expression is a fundamental aspect of animal development and a critical first step in bilaterian body plan organization [1, 2]. Within these domains, differential gene expression determines the functional properties of cells. For example, alternating domains of *pair rule* gene (e.g. *fushi tarazu*, *even skipped [eve]*) expression in the early Drosophila embryo organize the segmented body plan [3, 4]. In vertebrates, segmentally-organized somites form muscle segments and the vertebrae of the backbone [5–7]. In both cases, cells acquire their segmental identities along the anterior-posterior (A-P) axis through the functions of *Hox* genes. Further anteriorly, *Hox* paralogue groups 1–5 specify segments of the hindbrain (rhombomeres) [8–10]. How these segmented domains form has been the subject of intense investigation.

Morphogen gradients control the formation of segmental domains of gene expression, specifying distinct domains in a concentration-dependent manner. In the Drosophila embryo, maternal gradients of *bicoid* and *caudal* promote expression of different gap genes [11–15]. In vertebrates, secreted signaling molecules such as Fibroblast growth factor 8 (FGF), Wnt3a, and retinoic acid (RA) form gradients that influence somite formation [16–20]. Similarly, in the developing hindbrain, a network of FGF, Wnt and RA induce differential expression of Hox genes and Krox20 in adjacent rhombomeres [20–28]. However, morphogens are unlikely to be the only mechanism controlling segmentation in each of these cases. In particular, cell rearrangements are known to play a role. Steinberg’s *differential adhesion (DA)* hypothesis *(DAH)* predicts that cell sorting can generate distinct cell aggregates [29]. This mechanism works particularly well if cells of adjacent segments differ in the number or type of adhesion proteins they express, such as E-cadherin [30]. Similarly, contact-mediated repulsion can promote sorting. Repulsion between cells expressing *Eph* and *Ephrin* receptors is required for proper boundary formation between segments in both somites [31, 32] and rhombomeres [33–36]. When these two surface receptors come into contact, they elicit bi-directional signaling that causes cells to repel each other [37].

Wolpert’s classic French flag model posits that morphogens form well-defined gradients and that cells can precisely read the level of the signal at their location [38]. However, like any biochemical signal, morphogens are inherently noisy and the process of transducing the signal is stochastic. Noise can lead to mis-specification of cells, which will in turn produce a rough transition region between segments where multiple cell types co-exist in a salt-and-pepper arrangement. Actin cables or other physical barriers form at the interface between tissues in systems such as developing germ layers in early embryos [39], the Drosophila embryonic epidermis [40], or the zebrafish hindbrain [41] (reviewed in [42]). Thus, it is paramount that transition regions sharpen prior to the formation of these structures. The question thus becomes, how can a morphogen-organized system cope with stochasticity and generate refined, segmented zones of different cell types.

Cells may physically sort but the effectiveness of sorting is unclear, particularly in cases involving relatively small numbers of cells. For example, in the embryonic zebrafish hindbrain, rhombomeres are comprised of tens to a few hundred cells, depending on the stage [33, 34]. Very few cells occupy the local region near the interface between segments. The DAH assumes that tissues are liquid-like cell aggregates and that a sorted state is achieved as the system minimizes a tension/adhesion free energy. This however is primarily valid at macroscopic scales with large cell numbers [29, 30]. Furthermore, tissues do not necessarily behave as immiscible fluids [43]. Thus it is important to determine the effectiveness of cell sorting at smaller scales where the macroscopic assumptions of the DAH are not necessarily valid.

Previous work in the zebrafish hindbrain suggests that while cell sorting is important [33, 34, 36, 40, 44], cellular plasticity (e.g. transcription of target genes–*hoxb1a* and *krox20*) in response to morphogens (e.g. RA, Fgf and Wnt) also promotes sharpening of segment boundaries [45, 46]. Here, we use computational modeling to investigate the influences of these two different mechanisms. Since it involves both mechanical (e.g. adhesion, repulsion) and biochemical (e.g. gene transcription) processes, we develop a multi-scale model that accounts for both. Using this framework, we investigate each mechanism individually as well as in combination with others to determine effectiveness and potential interactions. Agent-based models treat each cell as a discrete entity with dynamically evolving properties [47, 48], while the Potts/Glazier-Graner-Hogeweg (GGH) model [49, 50] uses a lattice-based approach to account for dynamically evolving cell shapes [51] and cell-cell interactions. We use a sub-cellular element method (SCEM), which is similar to GGH, but allows more explicit descriptions of forces arising from cell-cell interactions [52, 53]. Each cell is treated as a collection of elements that interact according to user-defined forces. This has been used successfully to study the dynamics of epithelia [52, 53], the influence of Notch signaling on cell division [54], and homeostatic regulation in intestinal crypts [55]. We use SCEM to build a multi-scale, stochastic model of rhombomere boundary sharpening and investigate the effectiveness of cell sorting and plasticity (based on a stochastic description of *hoxb1a and krox20 in cells)*. We show that adhesion, repulsion, and plasticity all have a role in this process, none of which sharpens boundaries efficiently on its own. Instead, each has benefits and weaknesses, which are complementary and appear to work synergistically to accomplish this goal.

## Results

### Multiscale and stochastic models for cell sorting and plasticity

**Fig 1:**
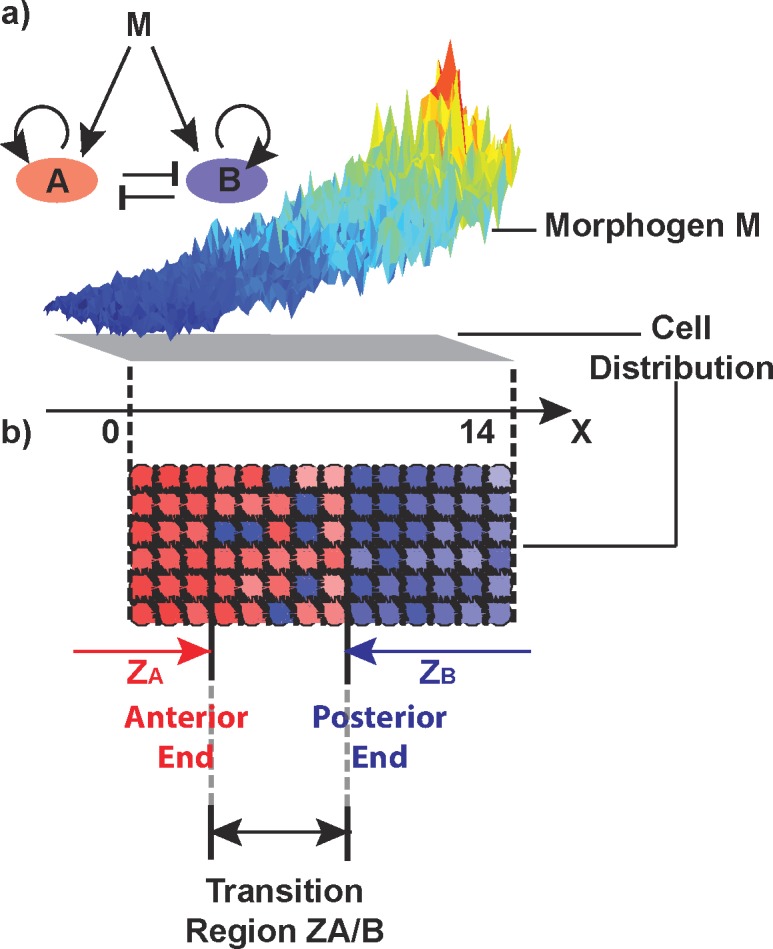
Model Schematic. a) Morphogen M in two spatial dimensions influences fate decisions of cells governed by a two-gene (A and B) circuit. A and B each promote their own expression and mutually inhibit one another. The morphogen, which is graded from high levels (red) posteriorly to low (blue) anteriorly, is modeled as noisy as are the internal genetic processes that transduce that signal. The global profile of the morphogen is given in Fig I in S1 Text. b) Morphogen M generates an initial transition region (5 cell diameters along the anterior-posterior axis) between an anterior gene A expression domain (ZA) and a posterior gene B expression domain (ZB), where cells expressing gene A or B (or some combination of both) are intermingled in a salt-and-pepper fashion.

How do distinct gene expression domains form in response to noisy positional information (Fig 1)? To address this question, we developed a set of hybrid computational models to investigate the effectiveness of different mechanisms at refining gene expression boundaries. Three possible mechanisms were considered: 1) differential adhesion, 2) cellular repulsion, and 3) cellular plasticity in gene expression.

We constructed three models–Sorting (S), Plasticity (P), and Sorting + Plasticity (SP). Model S includes only mechanical interactions such as cell-cell adhesion and repulsion. Model P assumes cells are stationary but allows for plasticity-mediated changes in cell fate. Model SP combines both. We used a discrete stochastic model formulation to account for low cell numbers. An SCEM framework endows each cell with a size, stiffness, and deformability (Fig A in S1 Text). The foundations of this method have been explained previously [52, 56]. To describe the influence of morphogens and gene regulation on cell identity, we constructed a spatial stochastic model of cell fate regulation. For each model, we utilized similar computational domains and initial conditions to aid direct comparison of results produced by each set of analyses. Motivated by rhombomere formation in the zebrafish, we consider a simplified computational domain consisting of a rectangular array of 6 cells in height with varying widths (Fig 1). We note that while this is a simplification, this dimension is on a similar scale in the horizontal direction; in the vertical direction, increasing cell numbers is computationally intensive yet does not give further valuable results, and thus we considered this simplified scenario. In most of our simulation, we compute our simulations in a time window corresponding to 10.7 to 12.7 hours post fertilization (hpf), during which the zebrafish rombomere 3/4 (r3/4) and 4/5 (r4/5) boundaries are sharpened [45].

The models discussed herein are necessarily complex. We thus focus on their aspects that are most relevant to this discussion. Specifically, we will consider how effective each is at sharpening gene expression boundaries, and where there are deficiencies, we will assess the source of that deficiency. Where possible, we will consider the sensitivity of results to model details. However, we note that given the complexity of these models, an exhaustive sensitivity analysis is not possible. We thus leave a detailed discussion of the sub-cellular element model that is the basis of Model S and the gene expression model that is the bases of Model P for references provided herein. Instead, we focus on the qualitative properties of these mechanisms and how they operate individually and in combination.

#### Model S

Model S incorporates only mechanical cell-cell interactions, specifically adhesion and repulsion, as a means of cell sorting. It omits cellular plasticity by ignoring the gene regulatory network (Fig 1). To describe individual cells, their interactions, and the forces that drive mechanical sorting, we utilized the SCEM (Fig A in S1 Text). In this computational formalism, individual cells consist of sub-cellular elements and interact according to a prescribed intercellular force potential. This force acts between every pair of elements within a cell, is repulsive at short ranges (to ensure elements remain separate and maintain an associated “volume”) and attractive at longer ranges (to ensure they retain a coherent structure). Given the elements that form cells in this formalism and the forces that connect them are not any direct representative of biophysical entities, we have chosen these intra-cellular forces so that in the absence of external forcing, the collection of elements that comprise an isolated cell round up to form a circular structure.

For these models, the manner in which cells physically interact is of critical importance. This model assumes that cells are one of two distinct types, where cells of the same type have a short-range attraction (adhesion) and cells of different types locally repel each other. While intra-cellular forces only act between elements of the same cell, inter-cellular forces act only between pairs of elements in different cells. If those elements are from the same cell type, the force is attractive at short range, and if they are from different cell types, it is repulsive. Additionally, to account for the contact mediated nature of forces in this model, all forces between pairs of elements are limited to a distance of two characteristic cell diameters (see supplementary material S1 Text section S1). For simplicity we will assume that there are two types of cells in this system (A and B). The attractive forces between two like cells is chosen to be the same independent of whether they are A-A or B-B interactions. These along with the forces between different cells (A-B interactions) were chosen to be strong enough to ensure cells meaningfully interact without introducing numerical artifacts that arise when forces become too strong.

**Fig 2:**
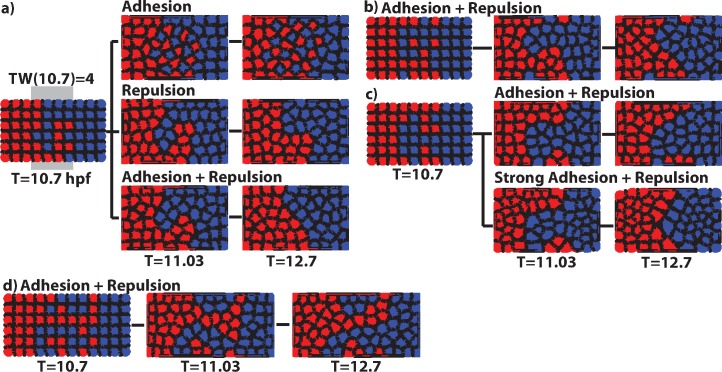
Performance of the mechanical cell-sorting model (Model S). a) Simulations incorporating adhesion only, repulsion only, and both. All simulations start from the same initial distribution (T = 10.7 hpf), all time units are hpf. Also see S1–S3 Movies. b) Example simulation where cells become isolated. c) Example where an aggregate of cells (a “clog”) becomes isolated and sharpening fails. When the strength of adhesion and repulsion interactions is strengthened, the end result improves. d) When the initial transition region is wide, cells are more likely to become trapped and a clear boundary does not form. Also see S4 Movie. These are snapshots of individual simulations from the data set in Table 1. See Table D in S1 Text for parameters.

In the absence of a gene regulation model, initial conditions for Model S are generated manually (rather than by a morphogen) by assigning a 3 × 6 zone of cells of one fate (red), a 3 × 6 zone of cells of another fate (blue), and a transition region of size (*N*−6) × 6 in between, where half the cells are randomly assigned blue and red fates respectively (Fig 2A). After specification of the initial condition, all cells are assumed to be unable to transition between different states and the only way they can self organize (outlying red or blue cells join other cells of the same color–Fig 2) is through movement. The domain length *N* varies to mimic wider or narrower initial transition regions. The strength of both adhesion and repulsion is modulated to determine the relative influence of each in the sharpening process. In all future simulations, we use the width of the transition region at time T (denoted by TW(T)) to measure the region sharpness. For example, the initial (T = 10.7 hpf) transition region shown in Fig 2A is TW(10.7) = 4, where space has been scaled so that one unit is a characteristic cell diameter.

#### Model P

Model P incorporates only gene regulation and plasticity-mediated cell fate transitions. It omits mechanical forces or any cell movements. The regulation in this model is an extension of [45], which is informed by zebrafish rhombomere specification (Fig 1). This model represents a gene regulatory system where *hoxb1a* and *krox20* genes are induced by a graded RA signal and mutually antagonize one another[28, 57, 58]. While the motivation comes from this specific context, the elements involved in this system—a morphogen M (representing RA in zebrafish rombomeres) and mutually antagonistic regulators A and B (representing *hoxb1a* and *krox20* in zebrafish rombomeres)—are common in other cell types/tissues. In this model, a noisy morphogen signal combined with stochastic responses leads to the formation of multiple coherent zones of different gene expression separated by noisy transition regions (Fig 1).

The morphogen is modeled as a diffusible molecule whose dynamics are encoded in a stochastic partial differential equation:

$$\frac{\partial {\left[M\right]}_{\text{out}}}{\partial t}=D\Delta {\left[M\right]}_{\text{out}}\underset{f_{1}}{\underset{︸}{-\left(1+\beta \right)k_{M}{\left[M\right]}_{\text{out}}+k_{M}{\left[M\right]}_{\text{in}}}}+\underset{\text{Production}}{\underset{︸}{V_{M}(x,t)}}+\underset{\text{Noise}}{\underset{︸}{\eta_{\text{out}}\frac{\text{d}\omega_{\text{out}}\left(t\right)}{\text{d}t}}}$$

where [*M*]_out_(**x**,*t*) is the extracellular concentration of the diffusible morphogen and [*M*]_in_ is the intracellular concentration. Here, the first term is a standard diffusion term. *f*_1_ represents morphogen production and signaling dynamics (along with morphogen removal from the diffusible domain) with *k*_*M*_ denoting the rate of exchange of morphogen between intra and extra cellular forms, and *βk*_*M*_ denoting the degradation rate of extracellular morphogen. The final two terms encode the production rate and stochasticity in the morphogen, with *V*_*M*_(**x**,*t*) the *M* production rate at position **x** at time *t* (see equation S2.2 in S1 Text). We impose no flux boundary conditions on the Nx6 computational domain, and focus on the 0 ≤ *X* ≤ 14 region where plasticity could induce cell transition shown in Fig 1 (for further details of our treatment of the morphogen, see S1 Text section S9 and Fig I). After an initial transient period, this will lead to a steady state situation where morphogen levels fluctuate around a fixed spatial profile.

Each cell in the immobilized array is further endowed with the gene regulatory model in Fig 1A (see S1 Text section S2, equations (S2.3)-(S2.6)), which accounts for internal stochasticity of gene transcription. Briefly, this network forms a bistable system where the morphogen ([*M*]_in_) concentration determines expression of *A* and *B* depending on its levels. The system describing these dynamics is a set of stochastic ODE’s:

$$\begin{array}{l} \frac{\text{d}{\left[M\right]}_{\text{in}}}{\text{d}t}=\underset{f_{2}}{\underset{︸}{k_{M}{\left[M\right]}_{\text{out}}-k_{M}{\left[M\right]}_{\text{in}}}}\underset{\text{Degradation}}{\underset{︸}{-\left[S\left({\left[M\right]}_{\text{in}}\right)\right]}}+\underset{\text{Noise}}{\underset{︸}{\eta_{\text{in}}\frac{\text{d}\omega_{\text{in}}(t)}{\text{d}t}}}\\ \frac{\text{d}\left[A\right]}{\text{d}t}=\underset{g_{1}}{\underset{︸}{\frac{C_{A}{\left[A\right]}^{n_{A}}+\kappa_{A}{\left[M\right]}_{\text{in}}^{m}}{1+{\left[A\right]}^{n_{A}}+{\left[B\right]}^{n_{B}}+\kappa_{A}{\left[M\right]}_{\text{in}}^{m}}}}\underset{\text{Degradation}}{\underset{︸}{-d_{A}\left[A\right]}}+\underset{\text{Noise}}{\underset{︸}{\eta_{A}\frac{\text{d}\omega_{A}(t)}{\text{d}t}}}\\ \frac{\text{d}\left[B\right]}{\text{d}t}=\underset{g_{2}}{\underset{︸}{\frac{C_{B}{\left[B\right]}^{n_{B}}+\kappa_{B}{\left[M\right]}_{\text{in}}^{m}}{1+{\left[A\right]}^{n_{A}}+{\left[B\right]}^{n_{B}}+\kappa_{B}{\left[M\right]}_{\text{in}}^{m}}}}\underset{\text{Degradation}}{\underset{︸}{-d_{B}\left[B\right]}}+\underset{\text{Noise}}{\underset{︸}{\eta_{B}\frac{\text{d}\omega_{B}(t)}{\text{d}t}}} \end{array}$$

where *f*_2_ represents the morphogen signaling and degradation dynamics, which is associated with *f*_1,_ and *g*_1_, *g*_2_ describe the regulation of levels of A or B by the intra-cellular concentration [*M*]_in_. [*S*([*M*]_in_)] represents *M* degradation due to another intracellular signal *S* (see equation S2.6 in S1 Text). Since morphogen levels are noisy and gene regulation is stochastic, initial specification of cell fates will naturally lead to a salt-and-pepper transition region where cells of different fates are intermingled. Unlike in model M, cell fates are not fixed and cells can change their gene expression, in this case, the degree of plasticity (how easily they switch expression between red and blue) is determined by a cell’s location, morphogen levels, and internal stochastic dynamics of gene regulation.

#### Model SP

This model combines both models S and P to account for both mechanical cell-cell interactions and plasticity. In model S, the mechanical properties of each cell depend on its identity, which remains fixed over time, and the prescribed force interactions between pairs of cells. In this combined model, those mechanical properties are determined by gene expression, which is a continuous quantity that varies in time based on position. Thus motions can influence gene expression and vice versa, introducing potential dependencies. In particular, gene expression plasticity has two important roles here: 1) it can potentially induce cell fate transitions as in model P, and 2) the resulting expression levels, in turn, regulate mechanical properties of cells and their interactions with each other (see S1 Text section S3). This leads to feedback whereby moving cells respond to different morphogen levels. As with model S, the mechanical interactions between cells are encoded as forces in a SCEM framework. As with model P, each cell is endowed with an identical and independent copy of the regulatory system in Fig 1A (S1 Text section S2, equations (S2.3)-(S2.6)), which determines cell fate. This model will be used to interrogate the effectiveness of the combination of these processes in refining spatial gene expression domains.

#### Sharpness index

We define a *Sharpness Index (SI)* to quantify the effectiveness of each mechanism at sharpening the boundary. SI is a measure that takes into consideration both the degree of mixing of different cell types as well as the straightness of the boundary [45]. It is defined as the standard deviation of the distance (measured from cell center) to the midline of the transition region of all mis-located cells. In addition to using SI, we categorize sharpening results of each individual simulation into three categories: boundary formed, where a clear boundary forms dividing the two cellular zones; boundary nearly formed, where mostly a boundary forms with one or two cells mislocated; boundary failed to form (for details of how they are categorized, see S1 Text section S5). SI is used in relevant figures and these categorization results are presented in relevant tables for the different models.

### Cell sorting is only effective when transition regions are narrow

Model S was simulated under a range of conditions including varied levels of adhesion and repulsion between cells. With a rectangular array of cells, we considered multiple initial conditions in which we varied initial transition width (ITW = 2, 3, or 4-cell wide transition regions). We manually populated the transition region with a random array of the two cell types with precisely a half-half mixture, and performed an ensemble of simulations for each condition. By comparing either the number of boundary formed and boundary nearly formed simulations under different conditions, we found (Table 1) that mechanical cell sorting was effective when the initial transition region was narrow, especially in cases with stronger cell adhesion strength (see Morse potentials in Fig B in S1 Text). A substantial fraction of these simulations ended with boundary nearly formed rather than formed. However, most outlying cells were at the top/bottom edges of the boundary where they have fewer neighbors (due to the structure of the domain) and are subjected to weaker sorting influences. For wider initial transition regions (ITW = 3), sharpening was reduced and strongly dependent on the strength of sorting. For ITW = 4 and wider, mechanical cell sorting was ineffective at boundary sharpening, no matter how strong the sorting forces.

**Table 1:** Boundary sharpening of cell sorting, starting with different initial transition region width (ITW) and under mild/strong cell sorting.

ITW	Strength	Boundary Formed	Boundary Nearly Formed	Boundary Failed to Form
2	Mild (16 total)	4	12	0
	Strong (16)	9	6	1
3	Mild (16)	0	12	4
	Strong (16)	4	11	1
4	Mild (16)	1	5	10
	Strong (16)	4	4	8

ITW is measured in typical cell diameters, for example, ITW = 2 means the initial transition region is 2-cell diameters wide. For each value of ITW, we first performed 16 simulations with mild cell sorting, then started from the same initial distributions but with strong cell sorting (same repulsion strength but increased adhesion strength, Morse potentials shown in Fig Ba in S1 Text). Out of the total 16 simulations in each case, the number of simulations that end up with a sharp boundary, a nearly sharp boundary, and those fail to form a boundary were recorded. For details of how the end states are categorized please see section S5 in S1 Text.

Since both differential adhesion and repulsive interactions between cells can lead to sorting independently [29], we next assessed the relative influence of each (Fig 2). Simulations were performed starting with identical initial conditions and adhesion or repulsion was either attenuated or strengthened. Inclusion of both adhesion and repulsion led to effective boundary sharpening (Fig 2A, bottom; S1 Movie). Removal of repulsion disrupted sorting, leading to a transition region that not only did not sharpen, but in many cases actually expanded (Fig 2A, top; S2 Movie). In contrast, removal of adhesion led to contiguous boundaries between regions of cells, though the resulting boundaries were far from straight (Fig 2A, middle; S3 Movie).

In cases where boundary sharpening failed, individual cells (Fig 2B) or cell groups (Fig 2C, top) were isolated from their preferred zone, mainly at the top or bottom edges of boundaries, as discussed previously. Since the only sorting interactions in this model were physical cell-cell interactions, once cells became isolated they encountered an isotropic environment with nothing biasing their direction of motion. Increasing the strength of cell-cell interactions reduced the frequency of these events (Table 1; Fig 2C, bottom). However, since there is a significant random component to the isolation of these cells, optimizing the properties of cell-cell interactions only marginally improved the outcome. Additionally, the likelihood of cells becoming isolated strongly depends on initial transition region width (ITW) and its noisiness. This is the primary reason that Model S became increasingly ineffective as the ITW increased (Fig 2D, S4 Movie). These results show that both adhesion and repulsion are required for proper sorting, and that these mechanical processes are only effective in boundary sharpening if the ITW is relatively narrow.

### Cell plasticity effectively narrows wide transition regions but is less effective at refining boundaries

**Fig 3:**
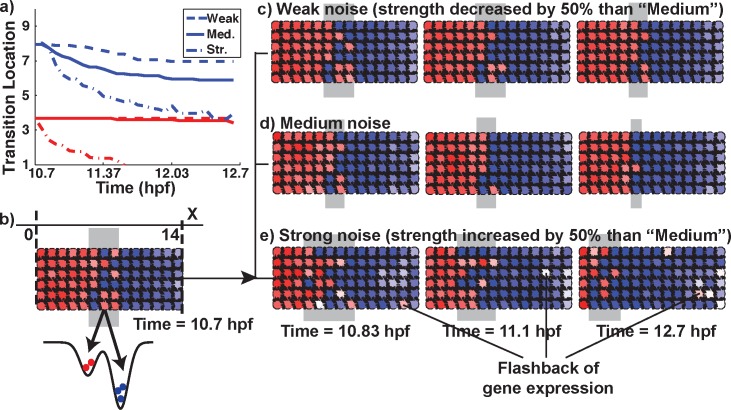
Performance of the plasticity model (Model P). a) Depiction of the average positions of the anterior and posterior edges of the transition zone over time for weak (dash line), medium (solid line) and strong (dash-dot line) gene expression noise strength. Results are averaged over 16 simulation replicates for each model. With medium strength, the boundary is clearly refined but is not fully sharpened. This refinement occurs as red cells in the transition region gradually change to blue, drawing the right side of the transition region toward the left. Weaker noise is less effective. Stronger noise leads to erroneous switching of cells from blue to red and the blue region expands at the expense of the red region. We note that all kinetic and morphogen parameters are the same here and only the noise amplitude changes. b) Simulation initial condition showing the results of initial patterning by the morphogen with a salt and pepper transition zone between anterior and posterior zones. The schematic “landscape” depicts bistability of the gene network, with a bias toward the “blue” fate (indicated by the relative depths of the two “wells”). c-e) Time-courses of gene expression noise-regulated boundary sharpening, starting from the same initial transition region (b), with weak (c), medium (d, also see S5 Movie) and strong (e) noise. The A-to-B gene switching proceeds from right to left. In the strong noise case, some cells revert their gene expression at the far right (posterior). See Table E in S1 Text for parameters.

With model P we asked how effective plasticity alone is at sharpening boundaries between cellular zones (Fig 3). If morphogen signals are noise free and gene regulation is deterministic, morphogens will always form precisely placed, sharp boundaries. In reality, however, this regulatory system is stochastic at every level. We have hypothesized that “noise-induced switching” helps sharpen rhombomeres in the zebrafish hindbrain [41]. This is based on the idea that while morphogen stochasticity introduces disorder near the boundary between cellular zones (i.e. a transition region), stochasticity in gene regulatory processes can also help cells to transition to the correct gene expression state [41]. To test this in our model, we omitted cellular motion so that sharpening relied solely on this mechanism.

In this model, initial cell fates were determined by a single morphogen, which was assumed to direct fate specification by influencing transcription levels of A and B (which for r3-5 of the hindbrain was modeled as *hoxb1a* and *krox20*). Upon application of the morphogen *M*, two expression domains formed with an intervening transition region (Fig 3B). Ensemble simulation results confirmed that the transition region partially sharpened after initial cell specification. When relatively little stochasticity (noise) in gene regulation was included in the simulations (gene expression noise strength *η*_*A*_ = *η*_*B*_ = 0.03, see equations S2.4, S2.5 in S1 Text), the transition region narrowed but did not sharpen (Fig 3C). Too much noise (*η*_*A*_ = *η*_*B*_ = 0.09) overwhelmed the system (Fig 3E). When moderate noise (*η*_*A*_ = *η*_*B*_ = 0.06) was included, however, sharpening was more effective (Fig 3D; S5 Movie). Fig 3A shows the average (across simulations) locations of the anterior (red) and posterior (blue) ends of transition regions as a function of time under different conditions. To ensure cell distributions have reached a steady state, we double the simulation time. Inspection of each of 16 replicate simulations shows that after T = 12.03 hpf cell fate transition rates drop and the system achieves a steady distribution of A and B cells (Fig M in S1 Text). These results confirmed that, for the medium noise case, the width of the transition region was reduced (but not completely) to about 2 cell diameters in width. This indicates that noise-driven sharpening can narrow an initially wide transition region, but is less effective at sharpening it completely.

It is also instructive to consider how this refinement occurs. Fig 3A shows that with moderate or weak stochasticity, sharpening in Model P occurred with the posterior edge of the transition region steadily moving toward a fixed anterior edge over time, reducing the region’s width. This results from an asymmetry in the underlying gene regulatory network that generates noise driven red → blue transitions (with the reverse much more rare). When noise levels are even higher, this red → blue transition was so prominent throughout the domain, that the posterior edge converged to the anterior edge and blue zone overtook the red zone over time (Fig 3E). This contrasted with mechanical sharpening (Model S), where red cells tended to move anteriorly and blue posteriorly, leaving a border mostly in the middle of the original transition region (at least when sharpening occurs)–though it also depended on the numbers of red and blue cells. This suggests that the cell switching from type A to B that occurs in Model P, but not S, leads to a fundamental difference in the directionality of boundary sharpening.

We make a final note about the role of stochasticity in promoting sharpening. The idea underlying the theory of noise-induced plasticity is that cell states (A and B) are represented by stable wells in an energy landscape (see Fig 3B for a schematic). Depending on the cells local environment (determined by position in our case), the relative depth (e.g. stability) of those wells may be different. In this context, for plasticity to aid sharpening, the “correct” state should be a deeper well and the incorrect a shallower well. In this way, an incorrect → correct (e.g. shallow to deep, see Fig 3B) transition would be more likely than the reverse. If the two wells are of roughly equal stability, both transitions would occur with equal likelihood, which would provide no benefit. Thus a sufficient level of asymmetry is required for noise to aid sharpening. Of course, if both wells were either too deep or too shallow relative to noise strength, stochasticity would either have no effect or overwhelm the system (illustrated in Fig 3E). Thus while stochasticity can provide a benefit, the system must be in an appropriate operating regime to take advantage of it.

### Cell plasticity and mechanical cell-cell interactions synergize in sharpening

**Fig 4:**
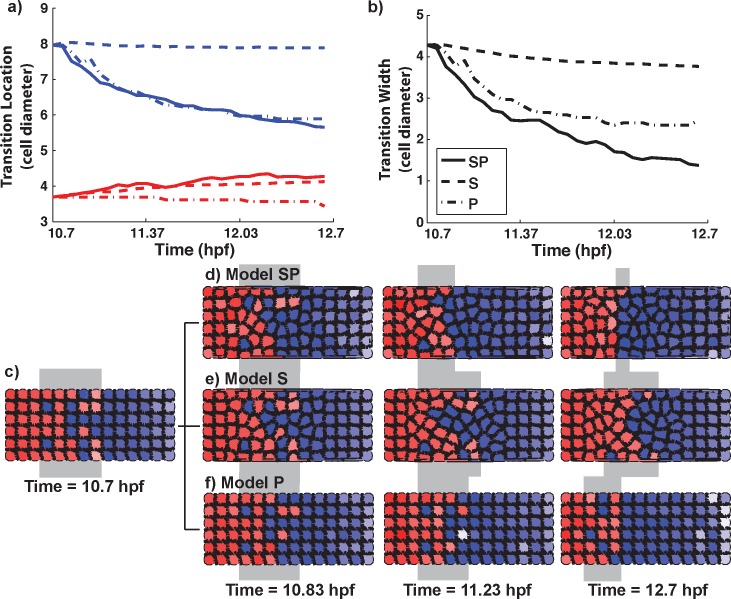
Combining mechanical cell sorting and noise mediated fate transitions is effective at fully sharpening the boundary. a) The locations of the anterior (red) and posterior (blue) ends of the transition region as a function of time, with both plasticity and cell sorting (solid lines), plasticity alone (dash-dot lines) and cell sorting alone (dashed lines). The results are averaged over 16 simulation replicates for each model. b) The transition width with both plasticity and cell sorting (solid lines), plasticity alone (dashed-dotted lines) and cell sorting alone (dashed lines). c-f) Time-courses of boundary sharpening under both plasticity and cell sorting (d, also see S6 Movie), cell sorting only (e) and plasticity only (f), starting from the same initial distribution (c). See Tables E and F in S1 Text for parameters.

**Fig 5:**
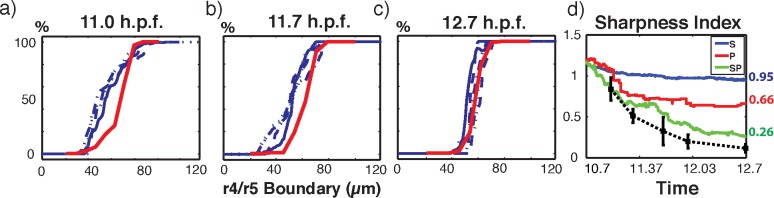
Comparison of model results with observations. (a-c) Comparison of Krox-expressed cell distribution near the r4/r5 boundary in zebrafish rhombomere (blue lines) with B cell distribution from model SP (red lines) at three time points. This figure indicates the percentage of cells that dominantly express Krox as a function of position along the segment. Experimental data are from [45], four samples are shown, each with one line style. Simulation results are the average of 16 simulations. d) Average SI over all 16 simulations from model S (blue line), P (red line) and SP (green line) compared with experimental data from zebrafish r4/r5 boundary sharpening (black dashed line with squares).

Our simulations with Models S and P show that mechanical sorting is effective at sharpening narrow transition regions while plasticity effectively narrows wider transition regions. How effective are these two mechanisms when combined? We hypothesized that plasticity narrows a transition region sufficiently to allow subsequent cell movements to complete sharpening. To test this, we considered the model SP, which essentially adds local cell-cell interactions that drive sorting to model P (Table 2; Figs 4 and 5D).

**Table 2:** Model comparison showing different levels of ZA/B boundary sharpening.

ZA/B	Boundary Formed	Boundary Nearly Formed	Boundary Failed to Form
S (16 Total)	1	8	7
P	5	7	4
SP	11	5	0
P followed by S T = 11.37 (11.7) hpf	5 (8)	10 (7)	1 (1)
S followed by P T = 11.37 (11.7)hpf	5 (5)	10 (10)	1 (1)

We started from the same 16 replicate initial conditions comprising a domain with zones ZA and ZB separated by a noisy transition region ZA/B between them (Fig 1). The three models under consideration, plasticity and cell sorting (SP), cell sorting alone (S), and plasticity alone (P) are each simulated to determine their efficacy. Additionally, model SP is simulated with the two mechanisms occurring simultaneously or in sequence. When in sequence, two times at which the activity of the two mechanisms change are tested (T = 11.37 and 11.7 hpf) to assess the influence of timing. All results are visually classified at T = 12.7 hpf.

Fig 4C–4F shows temporal snapshots of sorting, where each simulation begins from the same initial state, which is generated by the morphogen regulatory system (Fig 4C). These results provide a direct comparison of sorting resulting from plasticity alone (Model P, Fig 4F), mechanical sorting alone (Model S, Fig 4E), and the two combined (Model SP, Fig 4D, S6 Movie). In Model S, after the initial state is specified by the morphogen, the gene regulation system is turned off, and all cells are unable to alter their gene expression levels. Thus the morphogen system serves only to generate the initial condition for Model S. Results indicate that boundaries sharpen more effectively with SP than either S or P individually, based on tracking the average (over 16 simulations) position of the transition region borders and the transition region width (Fig 4A and 4B). With SP all simulations led to formed or nearly formed boundaries and a larger fraction formed completely (Table 2). Tracking the SI changes over time (Fig 5D) showing that model SP is the best among the three (end SI = 0.26), while model S is the worst that only reduces SI a little (end SI = 0.95), and model P sits in between (end SI = 0.66). The standard deviation of SI of the model S, P and SP is shown in Fig K in S1 Text. Tracking the boundaries of the transition region over time also revealed that rather than sharpening to either the center or one side of the transition region, the final boundaries were within the initial transition region but biased toward the anterior (Fig 4A). This is consistent with a combination of the two mechanisms ultimately driving sharpening. Additionally, simulation results indicated that the final location of the boundary was precise when the sharpening was driven by cell sorting and/or plasticity (see S1 Text section S7 for further details).

We next sought to determine if the order of action or duration of these different sharpening mechanisms influence the outcome. To do so, we performed numerical simulations (results in Table 2) where 1) plasticity was only active early, up to a pre-determined time point (T = 11.37 or 11.7 hpf) after which sorting became active, 2) the reverse, sorting was followed by plasticity, and 3) the two mechanisms occurred simultaneously and for the full duration of the simulations (i.e. the SP model discussed previously).

When sorting was active early and plasticity occurred later, outcomes (Table 2, “S followed by P”) were better than with sorting alone and comparable to plasticity alone, but still ineffective. Sorting followed by plasticity (“P followed by S’) on the other hand yields a substantial effect (Fig L in S1 Text). This indicates that the early action of plasticity followed by later action of sorting improves outcomes over either mechanism alone. The combination of the two (model SP) acting in concert for the full sharpening window however yields yet further improvement (Fig L in S1 Text). Combined, these results suggest that the two mechanisms, mechanical sorting and plasticity, can work synergistically with plasticity serving to narrow transition regions to a manageable width and sorting serving to finalize the sharpening process.

#### Comparison of simulation results with experimental data

We compare our model results with experimental data from [45]. In the zebrafish rombomere, an initial r4/5 transition region is typically ~40 μm in length along the anterior-posterior axis and later reduced to 5–10 μm which is ~1 cell diameter [45]. If we take the estimation 1 cell diameter ~ 8 μm, this information suggests the r4/5 boundary sharpens from ~5 to ~1 cell diameter, which our simulation results compare well to: within the time window from 10.7 to 12.7 hpf, the average transition width drops from ~4.28 to ~1.38 cell diameters, comparing to an end width of ~3.77 for model S and ~2.46 for model P (Fig 4B).

Next we inspect the B cell (corresponding to Krox-expressing cells in zebrafish rombomeres) distribution at different time point. Fig 5A–5C show the fluorescence measurements of Krox20 at 11, 11.7, and 12.7 hpf, with the four blue lines of different styles representing four different experimental samples, which we reproduced from [45]. These data are taken from a ~120 μm region along the anterior-posterior axis of the zebrafish rombomere that contains the r4/r5 transition region. Our simulation domain corresponds to an approximate window 20–100 μm centered in this experimental region. Fig 5A–5C show that the approximate transition width at the three time points are similar between data and simulation results. Further, at the end of the simulation, the density profile as a function of position for the simulations are comparable to observations. There are some small differences between model results and observations at earlier time points (Fig 5A and 5B). We note however that the model has not been tuned to match these distributions. Rather we have combined the gene expression model from [45] with a model of cell motions and find that results nearly match observations.

Finally we compare the time evolution of the SI between simulation results and experimental data of zebrafish rhombomere r4/r5 boundary (data taken from [45]). Fig 5D shows the experimental data of SI in the time window 11 to 12.7 hpf (black dashed line with squares), together with the average SI of models S (blue line), P (red line), SP (green line), respectively. This comparison clearly shows that the hybrid SP model most closely matches the sharpening data from experimental observations.

#### Relative strength/timescales of plasticity and cell sorting

Here we investigate how the strength and/or timescales of cell sorting and plasticity influence the sharpening process. Given the inability to measure and precisely parameterize these aspects of the model, this serves as a partial sensitivity analysis. Rather than perform a sensitivity analysis with respect to each parameter of the model, we assess sensitivity with respect to the strength of the critical features of the proposed mechanism, namely the strength of plasticity and sorting.

**Fig 6:**
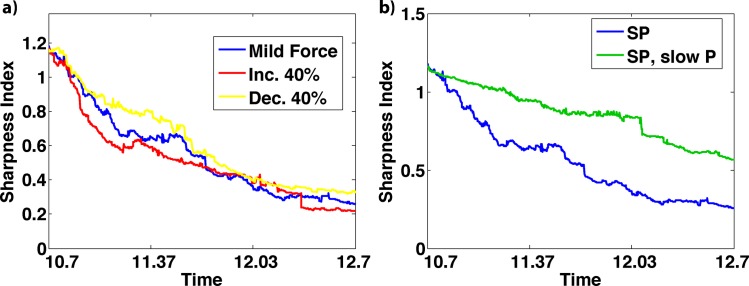
Assessing the influence of sorting and plasticity strengths on sharpening. a) Measuring the influence of cell-cell interaction strengths (which influences the sorting process) on sharpening. Results show the time dependence of the SI for different interaction strengths: mild cell sorting (blue line, this force strength is the same as used previously), interaction strengths increased by 40% (red line) and decreased by 40% (yellow line). Stronger interaction forces speed up sorting process while weaker interactions slows it down. The magnitude of the forces however has little effect on the final sharpness. b) Analysis of the influence of plasticity properties on sharpening. The blue / green curves depict the SI as a function of time for the normal plasticity model (the same as used in previous simulations) and with gene expression dynamics slowed down to impair cell fate transitions (also see S7 Movie).

In model SP, we first assess the influence of the cell sorting strength while keeping the plasticity component of the model unchanged. Results (Fig 6A) show that stronger cell-cell interactions lead to quicker sharpening. This is to be expected since faster cell speeds, which would be associated with stronger cell-cell interactions, should lead to quicker sorting of cells under the differential adhesion hypothesis. We note however that the effect here is minor. Thus fairly large changes in the cell-cell interaction properties have a relatively minor influence on the sharpening process. In other words, the sharpening process is insensitive to the precise details of cell-cell interactions.

Next, we keep the cell sorting strength fixed but slow down the plasticity induced gene expression switching. This is accomplished by introducing a time scale parameter into the gene regulatory kinetics that can be used to change the speed of the kinetics without changing the steady states of the system (see S1 Text section S2 for more details). Results clearly show that slowing down the gene regulatory kinetics leads to much slower and possibly incomplete sharpening (Fig 6B, S7 Movie). This is consistent with our previous results indicating that plasticity serves to narrow transition regions while sorting, which is only effective for narrow transition regions, serves to complete the process. Slowing the initial narrowing process renders the sorting process ineffective. Combined, these results support the conclusion that sharpening results from a combination of global (plasticity) and local (sorting) processes working synergistically.

### Simultaneous sharpening of two boundaries separating three domains

The zebrafish hindbrain consists of 7 rhombomeres. While these segments utilize different signals and potentially different mechanisms to form and sharpen, the RA morphogen along with the Hoxb1/Krox20 regulatory system are vital to the formation of rhombomeres 3–5 (r3-r5) (Fig 1). Up to this point, we have modeled sharpening between two domains, but we now consider how effectively cell movements and plasticity (Models S, P, and SP) are at forming and sharpening three cellular zones.

**Fig 7:**
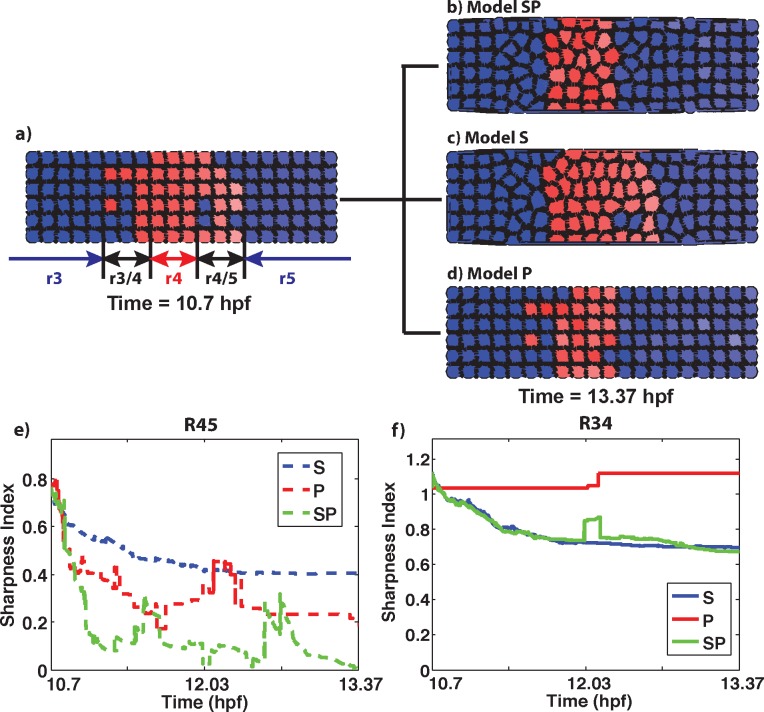
Representative simulation showing formation of three zones, indicative of rhombomeres r3-5 in the developing zebrafish hindbrain. a) Simulations of initial conditions depicting the gene expression domains (rhombomeres—r3, r4 and r5) and the transition regions (r3/4 and r4/5) between them. We use r3-5 notation rather than Za, Zb to identify these zones with rhombomeres. b-d) The final states of boundary sharpening with plasticity and mechanical cell sorting (b), mechanical cell sorting alone (c), and plasticity alone (d), all starting from the same initial condition. Also see S8 Movie. See Tables E and F in S1 Text for parameters. Panels (e,f) show the evolution of the SI for the r45 and r34 boundary respectively for each model.

To initialize the domain, the simulated RA morphogen generates a 20x6 domain of cells (Fig 7), which is similar in scale to the horizontal dimension of the r3-r5 zones at the onset of hindbrain specification in zebrafish [45]. We scaled the morphogen system such that the readout of the Hoxb1/Krox20 gene regulatory system in response to the RA gradient generates three zones of roughly the same size with transition regions in between. All three models (S, P, and SP) were simulated and the dynamics of the two transition regions were tracked over time. The combined model (SP) was highly effective at sharpening the r4/5 boundary (Table 3, Fig 7A and 7B, S8 Movie). When comparing the three models, models S or P individually were not as effective as SP, as was the case earlier in the 2-domain models. However, compared to 2-domain models, all three models (S, P, and SP) appeared to be more effective in the 3-domain scenario (Tables 2 and 3, Fig 7E). This results from the fact that the length scale of the RA morphogen was reduced to generate a sufficiently steep gradient to produce three cellular zones. Since the width of transition regions depends on the relationship between noise in the morphogen and steepness of the gradient, the transition widths in all 3-domain models were narrower than in the previous 2-domain simulations.

**Table 3:** Different sharpening mechanisms at r3/4 and r4/5 boundaries during development of zebrafish rhombomeres.

r3/4	Boundary Formed	Boundary Nearly Formed	Boundary Failed to Form
SP (16 total)	5	6	5
S (16)	5	6	5
P (16)	0	8	8
r4/5	Boundary Formed	Boundary Nearly Formed	Boundary Failed to Form
SP (16 total)	16	0	0
S (16)	6	8	2
P (16)	10	6	0

All simulations started from the same 16 initial conditions for a domain containing r3-r5 (Fig 7A) with r3/4 and r4/5 transition regions separating the respective zones. Each of the three models SP, S, and P were simulated to test their efficacy.

The dynamics of the r3/4 boundary were however significantly different than r4/5. At this boundary, plasticity was completely ineffective at sharpening in any simulations (Table 3; Fig 7). This reflects the fact that the interplay between stochasticity and the underlying gene regulatory network depends on morphogen levels. At the r3/4 boundary, morphogen levels are too low for plasticity to induce any state transitions. Models S and SP at this boundary performed nearly identically–especially when we compare the SI changes (Fig 7F). Additionally, simulations of model S at each of these two boundaries performed nearly identically. These results suggest that either mechanical sorting is the only manner of sharpening at the r3/4 boundary or some alternative type of cellular plasticity (e.g. other morphogens, other gene regulatory networks) is required to sharpen this boundary, unlike r4/5.

### Alternative possible mechanisms

As we discussed above, contact-based cell sorting only appears to be effective at forming a sharp boundary when transition regions are narrow. This could of course be the result of sub-optimal cell-cell interaction parameters. However modulating the strength of inter-cellular forces does not improve the situation much (Fig J in S1 Text). Furthermore, simulations of r3-r5 formation, where two boundaries must form and sharpen, suggest that while sorting may be effective at one boundary, it is unlikely to be as effective at adjacent boundaries. Recent experimental studies suggest that long ranged cell sorting or chemotaxis may be very effective at forming a clear and sharp boundary [59, 60]. To investigate the effect of inter-cellular force range on boundary formation, we perform a similar simulation study with the same parameter values as in the mild adhesion and repulsion combination, but increase the effective range of cell-cell interactions to about 3 cell diameters (see S1 Text section S1) to mimic longer range chemotactic effects. With this addition, boundary formation becomes much more effective (Fig J in S1 Text): starting with ITW = 3, all 16 simulations ended up with a clear boundary formed, while with ITW = 4, 13 out of 16 simulations formed a clear boundary, the other 3 failing as a result of isolated cells that are pushed to corners of the domain.

The key observation from these and results above is that local, cell-cell contact based sorting forces appear to only be effective at sharpening domain boundaries if the initial imperfections are confined to a narrow band. Our prior results indicate that plasticity based effects could improve this process by narrowing initially broad transition regions. This is not however the only possibility. Results here indicate longer range chemotactic forces between cells could play a similar role. Alternatively, chemotaxis towards or away from an organizing center could provide the same benefits. While we cannot rule out any of these on the basis of the data available, we do note that the plasticity based mechanism discussed above would yield qualitatively different predictions than these chemotaxis based mechanisms. The essential failure of the simple, contact based sorting process is its locality. Each of these additions provides a separate means of providing more global information to the cells. In the case of chemotactic mechanisms, that global information would drive longer length scale motions of cells. With the plasticity based mechanism on the other hand, narrowing of transition zones would arise from fate transitions rather than longer length scale motions. Thus the qualitative movement patterns of cells could provide a means to delineate these mechanisms in future experimentation.

## Discussion

Embryonic segments are fundamental building blocks of the body plan of many animals. While intense research has been directed at elucidating how segmentation occurs [1–10], it remains unclear how the borders between different segments sharpen. For example, in any morphogen signaling system that controls segmentation (such as bicoid or RA), noise in both the morphogen itself and in the transduction of the morphogen signal may reduce precision in the ability of responding cells to form sharp boundaries between segmental domains, which could have long lasting effects on development.

To date, multiple theories for boundary sharpening and maintenance have been proposed. Mechanical structures such as actin cables have been posited to generate tension that robustly separates segments [40, 42]. These structures however could be a double-edged sword. While they might help maintain a boundary once it is established, the presence of noise could impair their development, potentially leading to a permanent inability to sharpen further. Thus, a relatively well-defined boundary should be present before the formation of these structures. The “Differential Adhesion” (DA) hypothesis [29] argues that mechanical cell-cell interactions promote segment formation and sharpening. This hypothesis is however most often discussed in the context of systems containing thousands of cells that can be thought of as an active fluid. It is thus unclear how effective DA may be when smaller numbers of cells are present, such as in the zebrafish hindbrain.

Our modeling results suggest that local, contact based adhesion/repulsion mediated sorting is only effective at sharpening narrow (approximately ≤3 cell diameters) transition regions between segments. This is because once individual cells or cell groups become isolated from their respective aggregate, the local nature of contact-mediated cell-cell interactions cannot provide sufficient information to direct cells to the correct location. In this sense, an initially wide transition region is meta-stable state and can only resolve through random movements over time. Thus, when small numbers of cells are present, purely local, contact based sorting (e.g. differential adhesion sorting) appears to be insufficient to guarantee robust organization.

There are a number of possible processes that could be layered on top of contact based sorting to improve sharpening. Motility based processes such as chemo-attraction or chemo-repulsion of like or different cells could provide additional, longer range information to coax cells toward the appropriate region of the domain [60]. Our results show that this is an effective mechanism. Chemotaxis toward or away from a pre-existing organizing center could serve a similar purpose (as suggested in [59]). Here we investigate an alternative possibility, the “cellular plasticity” theory, which argues cells can change their fate by up- or down-regulating the expression of critical genes [45]. This is fundamentally distinct from the motility based processes discussed above in that long range taxis information is not required. Here we show that this mechanism has its strengths and weaknesses, but that when combined with local, cell contact based sorting, the two work together synergistically to promote sharpening.

Plasticity-mediated sharpening can be explained from the standpoint of an energy landscape view (Fig 3B) of cell fate regulation [61, 62]. This theory posits that cell fates can be thought of as, loosely speaking, minima (or wells) in an underlying energy landscape. Each valley in the landscape is associated with a particular fate and thus multiple wells indicate multi-potency. However, cells do not prefer all of these local energy minima equally (i.e. they differ in stability). Different depths are associated with relative stability and the greatest minimum is typically associated with the “correct” fate. In this view, a cell in the wrong fate (based on its position) simply resides in a different, shallower energy well and stochasticity can drive the cell to the correct fate. The shallower the minimum, the more likely noise can lead to fate switching. Thus, cells at a significant distance from the presumptive boundary are quickly driven to the correct fate in our model by differences in morphogen concentration, narrowing the transition region. Once it is narrow, however, morphogen differences from cell to cell near the boundary become small and cells have a nearly equal preference for the two possible states (i.e. the two fates are represented by wells that have roughly the same depth). This process of course requires the landscape to have the appropriate structure, which can vary with the state of the system (represented by parameters). Our sensitivity results however suggest that the qualitative structure of the underlying landscape does not change dramatically as parameters are changed.

For this reason, plasticity-mediated sharpening is effective at rapidly narrowing a wide transition region, but is very slow at completing the sharpening process and in many cases fails to do so. Our results for cell-contact based sorting show the opposite. This mechanism is effective at sharpening narrow boundaries, but performs poorly for wider transition regions where cells are intermingled. Thus the two mechanisms have complementary strengths and weaknesses. When combined however, their strengths synergistically promote sharpening, plasticity narrows the transition region while cell contact based sorting completes the process.

Given that many segmented tissues (e.g. the zebrafish hindbrain) are comprised of more than two cellular zones, we further investigated the effectiveness of this joint mechanism at sharpening the boundaries between multiple zones of different cell types. We found that the two mechanisms have different roles at different boundaries. Specifically, while plasticity-induced sorting is effective at reducing the width of transition regions at the r4/5 boundary, it is ineffective at the r3/4 boundary, a result of different morphogen levels placing the system in a different dynamical regime of the underlying gene regulatory network.

Given the distinct strengths of plasticity versus sorting, plasticity is of most value early in the sharpening process while the primary value of sorting becomes apparent later in the process (though a model where they are jointly active for the entire sharpening time frame is most effective). We are not aware of quantitative, time-dependent gene expression data here for the hindbrain, but in the developing mammalian embryo, cell fate decisions have been found to be gradual with gene expression diverging over the course of hours and multiple cell divisions [63]. This gradual fate specification (if present here) could have two effects. First, the relative ease and frequency of stochastically driven cell fate transitions is related to the level of gene expression distinctiveness (or in other terms, the level of specialization) of the two fates. That is, the more distinct two cell fates are, the more difficult it becomes to stochastically drive transitions between them. Second, it is possible that the difference in adhesion / repulsion properties between multiple cell types is directly related to distinctiveness of those cell types (e.g. more distinctive cells exhibit stronger sorting). Combined, these would lead to a scenario where plasticity would be more active early while sorting would become active at later times. We note that Calzolari et al. [40] did not observe cell fate transitions during rhombomere formation. However, they focused primarily on stages when actin cables form between segments, which occurs near the end of the boundary sharpening process when plasticity effects would be less likely to be observed. Zhang et al [45] on the other hand report co-expression of *hoxb1a* and *krox20* posterior to the future r4/5 boundary, consistent with cellular plasticity contributing to sharpening of this rhombomere boundary.

The most direct test of the hypothesis that plasticity and sorting both contribute to this process would be to simultaneously track cell positions and gene expressions from the earliest stage of rhombomere formation. An alternative, more indirect test of how the sharpening process occurs would be to track the spatial location of the resulting boundary as a function of time. If sorting alone is at work, the final location of a sharpened boundary will be near the center of the transition region. Alternatively, if plasticity plays a major role, that final boundary will consistently form near one end of the preceding transition region. Thus, these two mechanisms could potentially be distinguished with a smaller number of experimental observations and without tracking of individual cells.

We make one final note about the potential differences between these mechanisms. It is reasonable to expect that in addition to forming well-delineated boundaries, developmental systems should strive to ensure different segmented domains are the appropriate size. For example, the size of one rhombomere relative to its neighbors should be consistent (e.g. reproducible) across different embryos at the same developmental stage. A sharpening process that relies solely on sorting (whether it be contact based and / or chemo-repulsion / chemo-attraction / chemo-taxis based) of cells will have an inherent level of imprecision since the relative number of cells initially allocated to each of the cell lineages, which is stochastic, determines the final location of a boundary. A plasticity-based mechanism however provides a built-in control that could improve precision (see Fig G in S1 Text) since the final location of the boundary will be primarily determined by the morphogen itself.

While our results provide a general framework for explaining how initially noisy boundaries generated by a morphogen sharpen to form distinct segments, they also raise new questions. How do multiple segments (e.g. the zebrafish hindbrain or the neural tube) refine all zones jointly? While plasticity is vital to the refinement of noisy boundaries, the dynamics of morphogen signal transduction and the influence of stochasticity are highly dependent on the local levels of morphogen, which depend on position. Furthermore, morphogens have a spatial range of action limited by rates of diffusion, receptor binding, and the fidelity of signal transduction. In light of this, it is important to ask, can the same mechanisms and machinery be used to sharpen [1] unwanted transition regions. In large tissues beyond the length scales over which morphogens can act, other signals must be involved. But what about on smaller scales (e.g. multiple rhombomeres composed of only tens of cells)? Addressing these questions and gaining a more comprehensive understanding of how more complex systems organize will require moving beyond this setting and considering the presence of multiple morphogens (e.g. RA and Fgf in the hindbrain for example [26, 64–67]), how they function in parallel, and how they potentially interact in signaling pathways.

## Methods

The modeling framework was based on a hybrid approach incorporating both noise-driven plasticity and sorting-driven mechanics. Plasticity was modeled by stochastic PDEs for the morphogens and several ODEs for the interaction among gene expression and the morphogen evaluated in each cell [45]. The equations were solved using an explicit finite difference scheme. The mechanics were modeled using SCEM, with forces included to describe both cell-cell adhesion and repulsion. For model and simulation details, please see supplementary material S1 Text.

## Supporting Information

S1 Text Supplementary information including modeling details, parameter values and additional modeling results are presented in the supplementary material S1 Text. (PDF)

S1 Movie Performance of the mechanical cell-sorting model including both adhesion and repulsion (Model S). Fig 2A bottom are snapshots from this video at T = 11.03 and 12.7 hpf.
(MP4)

S2 Movie Performance of the mechanical cell-sorting model including adhesion only (Model S). Fig 2A top are snapshots from this video at T = 11.03 and 12.7 hpf.
(MP4)

S3 Movie Performance of the mechanical cell-sorting model including repulsion only (Model S). Fig 2A middle are snapshots from this video at T = 11.03 and 12.7 hpf.
(MP4)

S4 Movie Performance of the mechanical cell-sorting model with wide initial transition region (Model S). Both adhesion and repulsion are included. Fig 2D are snapshots from this video at T = 10.7, 11.03 and 12.7 hpf.
(MP4)

S5 Movie Performance of the plasticity model with medium noise (Model P). Fig 3B and 3D are snapshots from this video at T = 10.7, 10.83, 11.1 and 12.7 hpf.
(MP4)

S6 Movie Performance of the combined model (Model SP). Combining mechanical cell sorting and noise mediated fate transitions is effective at fully sharpening the boundary. Fig 4C and 4D are snapshots from this video at T = 10.7, 10.83, 11.23 and 12.7 hpf.
(MP4)

S7 Movie Slowing down gene expression dynamics in plasticity impairs cell fate transitions. For more details, please refer to S1 Text section S2.
(MP4)

S8 Movie Representative simulation showing formation of three zones, indicative of rhombomeres r3-5 in the developing zebra_sh hindbrain. Fig 7A and 7B are snapshots from this video at T = 10.7 and 13.37 hpf.
(MP4)
