# Supplementary material for: Cell Sorting and Noise-Induced Cell Plasticity Coordinate to Sharpen Boundaries between Gene Expression Domains
Source: PLoS Comput Biol. 2017 Jan 30;13(1):e1005307. doi: 10.1371/journal.pcbi.1005307 (PMC5279720; doi:10.1371/journal.pcbi.1005307)
Supplement: S1 Text — (PDF) [file pcbi.1005307.s001.pdf]

Supplementary Material: Cell sorting and noise-induced cell plasticity coordinate to sharpen boundaries between gene expression domains

December 20, 2016

Qixuan Wang[1,2,*], William R. Holmes[4,*], Julian Sosnik[1,3], Thomas Schilling[1,3], Qing Nie[1,2]

[1]Center for Complex Biological Systems, [2]Department of Mathematics, [3]Department of Developmental and Cell Biology, University of California Irvine, Irvine, CA 92697

[4]Department of Physics and Astronomy, Vanderbilt University, Nashville, TN 37212

Corresponding Author: qnie@math.uci.edu

Contents

S1 Model S and sub-cellular element method **2**

S2 Model P **3**

S3 Model SP **4**

S4 Representation of cells **5**

S5 Sharpness Index and Mixture Index **5**

S6 Sensitivity analysis on model P **6**

S7 The transition region location is precise after boundary sharpening by any of the three models **7**

S8 Noise induces gene expression switching may cause the loss of cells expressing one identity. **8**

S9 Parameter values 8

S1 Model S and sub-cellular element method

Here we outline the details of the models discussed in the main text. Model S is designed to model cell movement resulting from cell-cell adhesion and repulsion. To model individual cells, we use the sub-cellular element method (SCEM) [1] (Fig Aa), where each cell is represented by N_{node} nodes. Nodes within the same cell are subjected to intra-cellular biomechanical forces determined by a Morse potential Φ_{intra}:

$$\Phi_{\text{intra}}(r) = U_{\text{intra}} \exp\Big(-\frac{r}{\xi_{\text{intra}}}\Big) - V_{\text{intra}} \exp\Big(-\frac{r}{\zeta_{\text{intra}}}\Big) \tag{S1.1}$$

where r is the distance between the two nodes. Inter-cellular forces are determined by another Morse potential Φ_{inter} acting between nodes of different cells. In model S, we assume that each cell can express either of the two identities (red/blue). Φ_{inter} applies to nodes from two cells of the same type (red - red, blue - blue) imparting an attractive (adhesion) force with a short range repulsion (to avoid overlap). Nodes from cells of different type (red - blue) are subjected to a repulsive force:

$$\Phi_{\text{inter}}(r) = \begin{cases} U^{\text{Atr}}_{\text{inter}} \exp\Big(-\frac{r}{\xi^{\text{Atr}}_{\text{inter}}}\Big) - V^{\text{Atr}}_{\text{inter}} \exp\Big(-\frac{r}{\zeta^{\text{Atr}}_{\text{inter}}}\Big), & \text{same type;} \\ U^{\text{Rep}}_{\text{inter}} \exp\Big(-\frac{r}{\xi^{\text{Rep}}_{\text{inter}}}\Big), & \text{different type;} \end{cases} \tag{S1.2}$$

The Morse potentials we used in model S are depicted in Fig B, with different adhesion/repulsion strengths. The motion of the i-th node of the n-th cell resulting from the combination effect of intra- and inter-cellular Morse potentials is determined by the following equation:

$$\mathbf{v}^{\Phi}_{n,i} = \eta_{n,i} - \nabla_{\mathbf{x}_{n,i}} \sum_{j\neq i}^{N_{\text{node}}} \Phi_{\text{intra}}\Big(\big|\mathbf{x}_{n,i} - \mathbf{x}_{n,j}\big|\Big) - \nabla_{\mathbf{x}_{n,i}} \sum_{m\neq n}^{N_{\text{cell}}} \sum_{j}^{N_{\text{node}}} \Phi_{\text{inter}}\Big(\big|\mathbf{x}_{n,i} - \mathbf{x}_{m,j}\big|\Big) \tag{S1.3}$$

where $\eta_{n,i}$ is the noise term, N_{cell} is the total number of cells and N_{node} is the total number of nodes in each cell, and $\mathbf{x}_{n,i}$ is the position vector of the i-th node of the n-th cell. Both intra- and inter-cellular Morse potentials are truncated after $r = 2$, where $r = 1$ is the typical diameter of a cell. This truncation ensures forces are cell-cell contact based and do not extend to longer spatial ranges.

The canonical SCEM based only on Morse potentials often behaves poorly for systems involving large-scale cell movement, during which cells might be squashed or even "explode". To avoid such technical problems, we add another pair-wise potential to nodes within the same cell to endow the cell with more structure. We first divide the nodes into two layers, each with the same number of nodes ($N_{\text{node}}/2$). For each pair of neighboring nodes in the same layer ($\mathbf{x}_{n,i} \sim \mathbf{x}_{n,i\pm1}$), and each pair of corresponding nodes in the two layers ($\mathbf{x}_{n,i} \sim \mathbf{x}_{n,i\pm N_{\text{node}}/2}$), we apply the following potential Ψ (Fig

Ab):

$$\mathbf{\Psi}(\mathbf{x},\mathbf{y}) = \mu\frac{|\mathbf{x}-\mathbf{y}|-l}{|\mathbf{x}-\mathbf{y}|}\Big(\mathbf{x}-\mathbf{y}\Big) \tag{S1.4}$$

Hence for the i-th node in the n-th cell, the velocity resulting from the potential Ψ should be:

$$\mathbf{v}^{\Psi}_{n,i} = \mathbf{\Psi}(\mathbf{x}_{n,i},\mathbf{x}_{n,i+1}) + \mathbf{\Psi}(\mathbf{x}_{n,i},\mathbf{x}_{n,i-1}) + \mathbf{\Psi}(\mathbf{x}_{n,i},\mathbf{x}_{n,i\pm N_{\text{node}}/2}) \tag{S1.5}$$

With such an enhanced structure, the motion of a node is determined by two parts:

$$\frac{d}{dt}\mathbf{x}_{n,i} = \mathbf{v}^{\Phi}_{n,i} + \mathbf{v}^{\Psi}_{n,i} \tag{S1.6}$$

where $\mathbf{v}^{\Phi}_{n,i}$ and $\mathbf{v}^{\Psi}_{n,i}$ are determined by equations ($S1.3, S1.5$), respectively.

S2 Model P

Model P, originally developed in [2], describes a plasticity based process that is hypothesized to aid rhombomere boundary sharpening in the zebrafish hindbrain. Morphogen M diffuses across the entire domain. The dynamics of the extra-cellular M concentration is described by the following equation:

$$\frac{\partial[M]_{\text{out}}}{\partial t} = D_M\Delta[M]_{\text{out}} + V_M(\mathbf{x},t) - (1+\beta)k_M[M]_{\text{out}} + k_M[M]_{\text{in}} + \eta_{\text{out}}\frac{\mathrm{d}\omega_{\text{out}}(t)}{dt} \tag{S2.1}$$

where $V_M(\mathbf{x},t)$ is the M production rate at position $\mathbf{x}$ at time t:

$$V_M(x,y;t) = v_M\tanh\Big(\frac{x-(x_f-10)}{\alpha_M}+1\Big) \tag{S2.2}$$

and ω_{out} is a white noise. When entering a cell, M activates either gene A or gene B, and the following system of ODE's is evaluated for each cell:

$$\begin{aligned}
\frac{\mathrm{d}[M]_{\text{in}}}{\mathrm{d}t} &= k_M[M]_{\text{out}} - \big(k_M + [S([M]_{\text{in}})]\big)[M]_{\text{in}} + \eta_{\text{in}}\frac{\mathrm{d}\omega_{\text{in}}(t)}{dt}, && \text{(S2.3)}\\
\frac{\mathrm{d}[A]}{\mathrm{d}t} &= \frac{C_A[A]^{n_A} + \kappa_A[M]_{\text{in}}^m}{1+[A]^{n_A}+[B]^{n_B}+\kappa_A[M]_{\text{in}}^m} - d_A[A] + \eta_A\frac{\mathrm{d}\omega_{\text{A}}(t)}{dt}, && \text{(S2.4)}\\
\frac{\mathrm{d}[B]}{\mathrm{d}t} &= \frac{C_B[B]^{n_B} + \kappa_B[M]_{\text{in}}^m}{1+[A]^{n_A}+[B]^{n_B}+\kappa_B[M]_{\text{in}}^m} - d_B[B] + \eta_B\frac{\mathrm{d}\omega_{\text{B}}(t)}{dt}, && \text{(S2.5)}
\end{aligned}$$

where $[M]_{\text{in}}$ is the intracellular M concentration, and $[A]$, $[B]$ represent the concentrations of gene A and B in the cell, respectively; ω_{in}, ω_A and ω_B are white noise; $[S([M]_{\text{in}})]$ represents M degradation due to another intracellular signal S:

$$[S] = \begin{cases} k_{\text{deg}}\dfrac{\gamma\big([M]_{\text{in}}\big)^n}{1+\gamma\big([M]_{\text{in}}\big)^n + f_0e^{-\lambda(x_f-x)}}, & \text{if } 0<x<x_f-10\\ k_{\max}, & \text{if } x<0 \text{ or } x_f-10<x<x_f \end{cases} \tag{S2.6}$$

In the simulations of slowed down plasticity induced transitioning (Fig 6b, Movie S7), a common factor 0.1 is multiplied to all other terms in the right hand side of equations (S2.1 - S2.5), with all noise terms multiplied by $\sqrt{0.1}$.

S3 Model SP

Model SP is a hybrid of models S and P, where cells are mobile and the intra-cellular gene concentrations can change due to motions of cells as well as stochastic effects, which in turn feedback to affect inter-cellular forces (which depends on gene expression). As in model S, cell movements are the result of inter-cellular adhesion and repulsion, which are determined by the cell's gene expression (levels of A and B, which depend on morphogen levels M). As a result of cell motions, concentrations of A and B and adhesion / repulsion properties of each cell are continuously changing, forming a feedback between gene expression and motility. To reflect this, unlike in model S where Φ_{inter} is a two-state Morse potential (equation ($S1.2$)), in model SP, Φ_{inter} is a continuous family of Morse potentials (Fig C):

$$\begin{aligned}\Phi_{\text{inter}}\big(\mathbf{x}_{m,j},\mathbf{x}_{n,i};\mathbf{g}^m,\mathbf{g}^n\big) &= U_{\text{inter}}\big(\mathbf{g}^m,\mathbf{g}^n\big)\exp\Big(-\frac{|\mathbf{x}_{m,j}-\mathbf{x}_{n,i}|}{\xi_{\text{inter}}}\Big) \\ &\quad -V_{\text{inter}}\big(\mathbf{g}^m,\mathbf{g}^n\big)\exp\Big(-\frac{|\mathbf{x}_{m,j}-\mathbf{x}_{n,i}|}{\zeta_{\text{inter}}}\Big)\end{aligned} \tag{S3.1}$$

where $\mathbf{x}_{m,j},\mathbf{x}_{n,i}$ are the position vectors of the two interacting nodes from two different cells: the m-th cell and the n-th cell, $m \neq n$. $\mathbf{g}^n$ is the gene expression concentration vector of the n-th cell, $\mathbf{g}^n = ([A_n],[B_n])$ where $[A_n],[B_n]$ are the concentrations of gene A and B in the n-th cell, respectively. Note that whereas the position of different nodes within a cell can be different, gene expression is a cell property and thus every node within the same cell is assigned the same gene expression. The adhesion and repulsion strengths between two cells (U_{inter} and V_{inter}) then depend on the gene expression of both cells in the following way:

$$U_{\text{inter}}\big(\mathbf{g}^m,\mathbf{g}^n\big) = \Big[1-\mathcal{F}\big(\mathbf{g}^m,\mathbf{g}^n\big)\Big]U_0 + \mathcal{F}\big(\mathbf{g}^m,\mathbf{g}^n\big)U_1 \tag{S3.2}$$

$$V_{\text{inter}}\big(\mathbf{g}^m,\mathbf{g}^n\big) = \mathcal{F}\big(\mathbf{g}^m,\mathbf{g}^n\big)V_1 \tag{S3.3}$$

where $\mathcal{F}(\mathbf{g}^m,\mathbf{g}^n\ ) \in [0,1]$ is a symmetric function of $\mathbf{g}^m,\mathbf{g}^n$ measuring the similarity of the gene expression in the two cells, which is constructed in the following way.

For the n-th cell, we linearly scale the concentration difference $[A_n]-[B_n]$ into a range of $[-1,1]$ and denote this scaled result as δ_n, so that $\delta_n = 1$ when the cell expresses maximum A and minimum B, and $\delta_n = -1$ when the cell expresses maximum B and minimum A. Then we define

$$\mathcal{F}\big(\mathbf{g}^m,\mathbf{g}^n\big) = \frac{1}{2}(\delta_m\delta_n + 1) \tag{S3.4}$$

When the two cells express the same gene in maximum amounts ($\delta_m = \delta_n = 1$ or $\delta_m = \delta_n = -1$), we have $\mathcal{F} = 1$ and equation (S3.1) gives the maximum adhesion potential (Fig C, red line); on the other hand, when they express the different genes in maximum amounts ($\delta_m = \pm 1, \delta_n = \mp 1$), $\mathcal{F} = 0$ and equation (S3.1) gives the maximum repulsion potential (Fig C, blue line). This quantity thus allows us to vary the strength of attractive and repulsive forces based on the similarity or differences between two cells.

S4 Representation of cells

In SCEM, each cell is represented by a collection of nodes (Fig Da). For visualization purposes however, it is helpful to depict cells as a confined area with a boundary. The two-layer structure of cells in this framework makes it simple to draw these cellular boundaries based on node positions. To visualize each cell, we assign the region enclosed by the outer layer of nodes as being part of the cell (Fig Db). This leaves a significant portion of the computational domain unassociated with each cell. However, since the zone of influence of each node extends a certain distance, we will assign part of the region between the outer node layers of adjacent cells as belonging to one of those two cells. To do so, we create a triangular mesh of the portion of computational mesh outside of the outer node layer of all cells using the outer layer nodes as vertices of the triangles (Fig Dc). Each resulting triangle can take one of two forms and its area can be apportioned to the adjacent cells accordingly.

1. One vertex belongs to one cell, and the other two vertices belong to another cell (Fig Dd). In this case, the triangle is associated with only two cells and we divide the triangle into one triangle and one quadrilateral separated at the midline of the original triangle. The resulting two areas are subsequently assigned to the two associated cells and colored accordingly.

2. The three vertices belong to three different cells (Fig De). In this case we divide the triangle into four smaller triangles by connecting the midpoints of each of the three sides of the triangle. The central triangle is unassigned (and uncolored) while the remaining three triangles are assigned to the respective cells and colored accordingly.

We treat all triangles from the mesh in this way, which gives the final representation of cells (Fig Df).

S5 Sharpness Index and Mixture Index

For each model simulation, we assign two measures of sharpness to quantify how well defined the resulting boundary between domains is.

The first measurement is the *Sharpness Index* (SI), which quantifies both the formation of a clear boundary and the straightness of the boundary. We adopt this index from [2]: SI is the standard deviation of the distance (measured from cell center) to the midline of the transition region of all mis-located cells. SI is evaluated over time for a simulation.

In addition to SI, to categorize the final state of a simulation, we introduce in another measurement: the *Mixture Index* (MI), which is evaluated at the endpoint of a simulation to quantify the degree of mixture of the transition region. MI is defined in the following way:

1. If a clear boundary forms dividing the two colonies, MI=0 (Fig Eab). That is, this index is intended to indicate the coherence of a boundary and does not quantify its straitness. Thus a boundary can be sharp but not strait.

2. If a cell is mislocated and at either the top or bottom of the computational domain, it contributes to MI a value equal to the shortest distance between itself and the presumptive boundary times 0.5 (Fig Ecd, mislocated blue cells at the boundaries).

3. If a cell mislocates so that it is separated from the appropriate grouping (e.g. a red cell surrounded by blue), it contributes to MI a quantity equal to its shortest distance to the presumptive boundary (Fig Ed, mislocated red cell).

We down-weight mislocated cells at the top/bottom since their location near the domain border leads to weaker sorting forces and thus in larger tissues, this effect would be expected to have less relative influence. In this way, each mislocated cell contributes to the calculation of the MI, with more mislocated cells leading to a larger value of MI. In short, this defines a weighted accounting of the number of mislocated cells to describe how sharp a boundary is.

Using the MI measure, we categorize sharpening results into three categories: boundary formed (MI=0), where a clear boundary forms dividing the two cellular zones; boundary nearly formed (MI$\leq$2), where mostly a boundary forms with one or two cells mislocated; boundary failed to form (MI>2). See Tables 1-3 for simulation results. While we distinguished between "boundary formed" and "boundary nearly formed" here, *in vivo* there is likely little distinction since subsequent formation of actin cables (or similar structures) could correct these final minor errors [3], or apoptosis of one or two cells could complete the process at relatively little cost. In Tables A - C we present the average end MI corresponding to simulation groups presented in Tables 1-3.

For future reference, the MI measures the degree of mixing of cells at the boundary whereas SI accounts for both the degree of mixing and how straight the border is. As a comparison example, we give both MI and SI of the end states of four simulations from model S in Fig E.

S6 Sensitivity analysis on model P

In this section we analyze how sensitive model P is to several of its parameters.

First, we find that model P is stable to parameters in the morphogen equations (S2.1, S2.3). Changes in the parameters D_M (the diffusion coefficient for extracellular M), β (which represents the relative strength of extracellular M degradation compared to cell uptake), and k_M (the rate of morphogen exchange between the cell and the extracellular medium), within a certain region will not affect the bistable switching property of the system, although they will cause some quantitative changes in the sharpening, as shown by Figs Fa-c: the average SI (out of 16 simulations) of different values of D_M, β, k_M behave quite similar.

On the other hand, parameters in the two gene equations (S2.4, S2.5) might change the system bistable switching property greatly. For example, we change the value of C_A in equation (S2.4), which equals 0.9 in all other simulations to create the $A \to B$ (i.e., red $\to$ blue) only transitions. With $C_A = 1$, we no longer see $A \to B$ transitions in the transition region, instead, there are reversed transitions (i.e., $B \to A$) happening in the more posterior region (Fig Fe); while with $C_A = 0.8$, lots of $A \to B$ happening in the whole observed region, resulting in all cells expressing gene B soon (Fig Ff). These effects are better illustrated by calculating the B cell distribution: with $C_A = 1$ (Fig Fg), the observed region suffers a loss of B cells while with $C_A = 0.8$, B Cells take over the whole region (Fig Fh). The average B cell distributions in the observed region (out of 16 simulations each) with $C_A = 0.9, 0.8$ and 1 are shown in Fig Fi, illustrating the differences of the bistable switching of the systems.

S7 The transition region location is precise after boundary sharpening by any of the three models

In addition to being sharp, the boundary location between segments must also be precise for robust and reproducible development. In this system, three important factors influencing precision: morphogen noise, the type of cell sorting, and gene expression noise.

Morphogen noise generates the salt and pepper transition region between zones. Thus, in a sense, the morphogen can be thought of as generating a noisy initial condition which subsequent dynamics serve to sharpen. In general, our simulations can be divided into three stages. First, an initial stage in which morphogen noise usually leads to an extremely wide initial transition region (Fig Ga). After this initial phase, we turn off the morphogen noise and let the system relax to reach a steady state, during which the early wide transition region naturally narrows from the initial right (posterior) side down to a narrow one typically of 3-6 cells wide (Fig Gb). This produces the initial condition from which simulations proceed. In general, this initial generation of zones leads to variability in the location of the left edge of the transition zone (Std = 0.96, Fig Gf). Interestingly, there is no variability in the location of the right edge (Std $\sim$ 0, Fig Gg).

We next assessed how the precision of the final location of zone boundaries evolve with time in models P, S, and SP (Figs Gc-e). While the mean of the locations have been discussed in the main text, here we focus on the variability, i.e., the precision. Results indicate that Model SP leads to the greatest reduction in the variability in the location of the left edge of the boundary, while the three models perform roughly the same at the right edge of the boundary. Thus, a model that combines both cell sorting and plasticity leads to the greatest precision in the location of domain boundaries.

S8 Noise induces gene expression switching may cause the loss of cells expressing one identity.

Plasticity induced red $\rightarrow$ blue transitions result in the loss of cells of one identity. Since the switching is one-way only due to the structure of the gene expression system, A $\rightarrow$ B in our model, this will certainly cause a loss in cells expressing gene A. When the domains are large, such a loss might not be a serious problem, yet for zebrafish rhombomeres which are initially low in cell numbers, it must be considered. We investigate two noise strength: mild (which is used in Fig 7) and strong, with noise strength $\eta_A = \eta_B = 0.06$ and 0.09, respectively. In our 16 simulations, initially (Time=10.7 hpf) the average number of A cells in the whole domain is ~ 39, with mild noise at the end (Time=12.7 hpf) the average A cell number is ~ 23 while with strong noise it is ~ 9(Fig Hab). That is to say, with mild and strong noise, gene A expression cells suffer a loss of 39.1% and 76.6%, respectively. When cell sorting is added to plasticity with mild noise, the cell loss phenomenon is slightly improved – at the end (Time=12.7 hpf) the average A cell number is ~ 25.5 (Fig Hc).

S9 Parameter values

Parameter values used in our simulations are listed in Tables D - F, where x and y are stated in units of cell diameters (cd). The truncation distance of Morse potentials in model S and SP is 2 cd. In most of our simulations, the computation time window is $0 \leq t \leq 3000$, which corresponds to the physical time window $10.7 \leq T \leq 12.7$ hours post fertilization (hpf).

For Figs 1, 3 and 4, the morphogen generates a domain of dimension 125 cd $\times$ 6 cd, and we only show the part 33 cd $\leq \overline{x} \leq$ 47 cd. This is the region where plasticity can induce gene identity transitions. For clarity, we place a new X-axis on this visualized portion of the domain with a range 0 cd $\leq X \leq$ 14 cd. The global morphogen is shown in Fig I, and the local morphogen in Fig 1a is the region between the two black lines in Fig I. For Fig 7, we compress the morphogen in the X direction, and instead it generates a domain of dimension 20 cd $\times$ 6 cd, in which case we show the whole domain in Fig 7.

References

[1] Newman, Timothy J. "Modeling multicellular structures using the subcellular element model." Single-Cell-Based Models in Biology and Medicine. Birkhuser Basel, 2007. 221-239.

[2] Zhang, Lei and Radtke, Kelly and Zheng, Likun and Cai, Anna Q and Schilling, Thomas F and Nie, Qing. "Noise drives sharpening of gene expression boundaries in the zebrafish hindbrain." Molecular systems biology, 2012. **8**(1).

[3] Terriente, Javier, and Cristina Pujades. "Cell segregation in the vertebrate hindbrain: a matter of boundaries." Cellular and Molecular Life Sciences 72.19 (2015): 3721-3730.

Table A Average end MI of boundary sharpening of cell sorting, corresponding to Table 1.

ITW	Strength	Average end MI
2	Mild (16 total)	0.88
	Strong (16)	0.28
3	Mild (16 total)	2.06
	Strong (16)	1.03
4	Mild (16 total)	5.81
	Strong (16)	6.69

Table B Average end MI of ZA/B boundary sharpening, corresponding to Table 2.

ZA/B	Average end MI
S (16 Total)	2.84
P	1.34
SP	0.22
P followed by S<br>T = 11.37 (11.7) hpf	0.75 (0.56)
S followed by P<br>T = 11.37 (11.7) hpf	1 (0.75)

Table C Average end MI of r3/4 and r4/5 boundary sharpening, corresponding to Table 3.

	r3/4 Average end MI	r4/5 Average end MI
SP (16 total)	1.16	0
S(16)	1.45	0.63
P (16)	3.16	0.5

Table D Parameter values used in model S. "cd" stands for "cell diameter".

Parameters	Values	Units
U_{intra}	6.5×10^{-3}	cd^2 sec^{-1}
V_{intra}	1.3×10^{-3}	cd^2 sec^{-1}
ξ_{intra}	0.375	cd
ζ_{intra}	0.45	cd
$U_{\text{inter}}^{\text{Atr}}$	2.6×10^{-3}	cd^2 sec^{-1}
$V_{\text{inter}}^{\text{Atr}}$	1.3×10^{-3}	cd^2 sec^{-1}
$\xi_{\text{inter}}^{\text{Atr}}$	0.625	cd
$\zeta_{\text{inter}}^{\text{Atr}}$	2	cd
$U_{\text{inter}}^{\text{Rep}}$	2.3×10^{-3}	cd^2 sec^{-1}
$\xi_{\text{inter}}^{\text{Rep}}$	0.4	cd
μ	2.08	sec^{-1}
l_{out} (outer layer)	0.28	cd
l_{in} (inner layer)	0.14	cd
l_{inter} (inter layer)	0.225	cd
dt	0.24	sec

Table E Plasticity parameter values used in model P and SP. Many are taken from [2] where the plasticity model was originally proposed. "cd" stands for "cell diameter".

Parameters	Values	Units
D_M	0.026	cd^2 sec^{-1}
β	1	—
k_M	8.3×10^{-4}	sec^{-1}
η_{out}	0	—
v_M	10.42	sec^{-1}
x_f	125	cd
α_M	15	cd
k_{deg}	208.33	sec^{-1}
γ	2.5×10^{-5}	—
n	1	—
f_0	400	—
λ	0.076	cd^{-1}
k_{max}	0.42	sec^{-1}
$C_A,\ C_B$	0.9, 1	—
$n_A,\ n_B$	2, 2	—
$\kappa_A,\ \kappa_B$	0.15, 0.15	—
$d_A,\ d_B$	0.4, 0.4	—
m	2	—
η_{in}	0	—
η_A, η_B	0.06	—
dx	0.25	cd
dy	0.25	cd
dt	0.24	sec

Table F Mechanical parameter values used in model SP. "cd" stands for "cell diameter".

Parameters	Values	Units
U_{intra}	6.2×10^{-3}	$\text{cd}^2\ \text{sec}^{-1}$
V_{intra}	2.6×10^{-3}	$\text{cd}^2\ \text{sec}^{-1}$
ξ_{intra}	0.3	cd
ζ_{intra}	0.45	cd
U_0	4.9×10^{-3}	$\text{cd}^2\ \text{sec}^{-1}$
U_1	1.2×10^{-3}	$\text{cd}^2\ \text{sec}^{-1}$
V_1	3.1×10^{-3}	$\text{cd}^2\ \text{sec}^{-1}$
ξ_{inter}	0.625	cd
ζ_{inter}	1.375	cd
μ	1.67	sec^{-1}
l_{out} (outer layer)	0.28	cd
l_{in} (inner layer)	0.14	cd
l_{inter} (inter layer)	0.225	cd
dt	0.24	sec

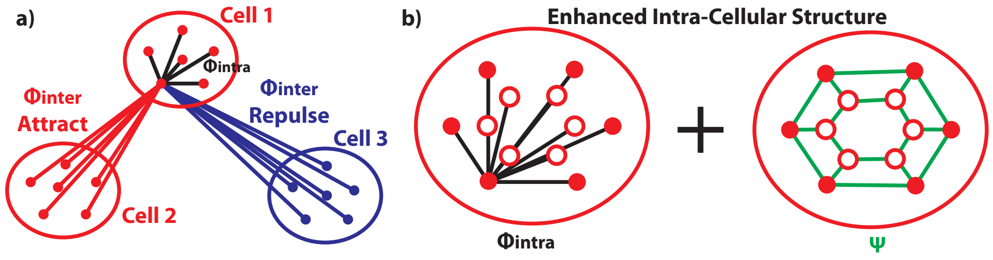

Fig A Illustration of SCEM implementation of cell-cell interactions in Models S and SP. a) Each cell is represented by a collection of nodes or elements. Nodes within the same cell are subjected to an intracellular Morse potential Φ_{intra}. Nodes from different cells are subjected to a two-state intercellular Morse potential Φ_{inter}, which either shows a short-range repulsion and a long-range attraction if the cells involved are the same type, or a long-range repulsion if the cells are different types. To simulate the contact-based cell sorting, all potentials are truncated after approximately two cell diameter. b) For the intracellular structure, in addition to the Morse potential Φ_{intra}, we add another potential Ψ. The nodes are evenly distributed in two layers, and Ψ applies to any pair of neighboring nodes within the same layer, and any pair of corresponding nodes from the two layers. This additional potential endows the cell with more stable structure.

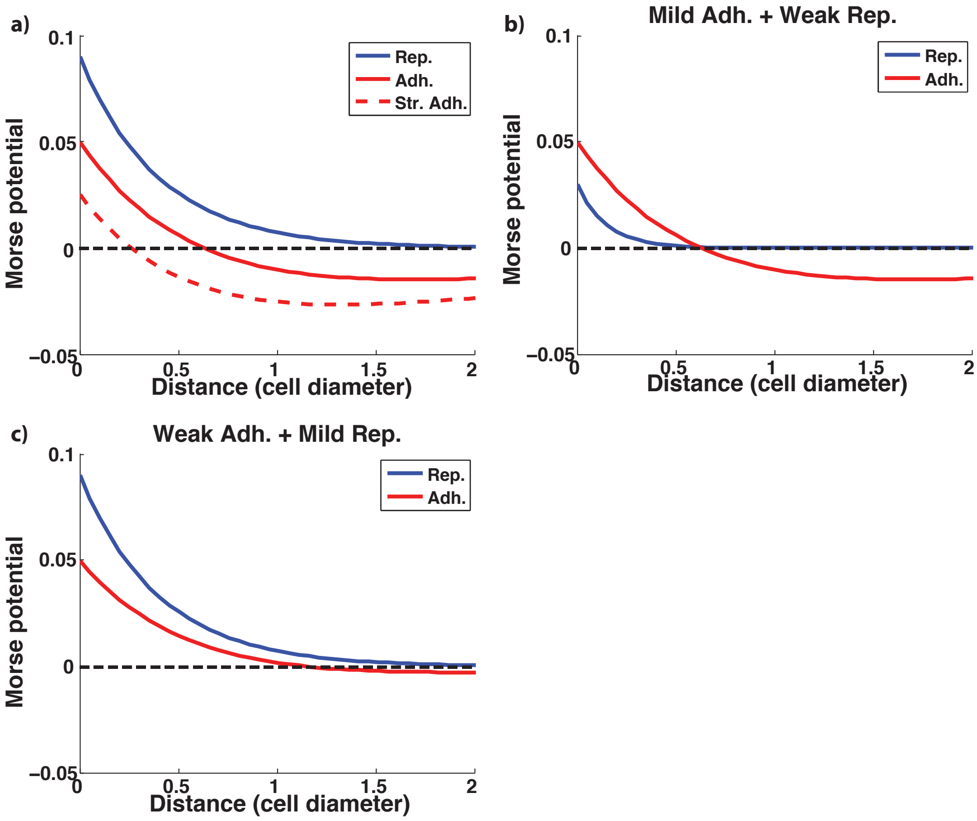

Fig B Inter-cellular Morse potentials for model S, linked to the model generating results in Fig 2. Parameter values for mild adhesion and mild repulsion (red and blue lines in Fig Ba) are given in Table D, other Morse potentials are resulted by changing a few parameter values, as discussed. a) Mild adhesion (red solid) and mild repulsion (blue solid) in Figs 2a-d; the strong adhesion (red dashed) and mild repulsion (blue solid) in Fig 2d. For strong adhesion (red dashed), all other parameter values are the same as in Table D except for $V_{\text{inter}}^{\text{Atr}} = 1.9 \times 10^{-3}$ cd^2 sec^{-1}. b) Mild adhesion (red) and weakened repulsion (blue) in Fig 2a. For weakened repulsion, $U_{\text{inter}}^{\text{Rep}} = 7.8 \times 10^{-4}$ cd^2 sec^{-1}, and $\xi_{\text{inter}}^{\text{Rep}} = 0.15$ cd. c) Weakened adhesion (red) and mild repulsion (blue) in Fig 2a. For weakened adhesion, $\xi_{\text{inter}}^{\text{Atr}} = 1$ cd. “cd” stands for “cell diameter”.

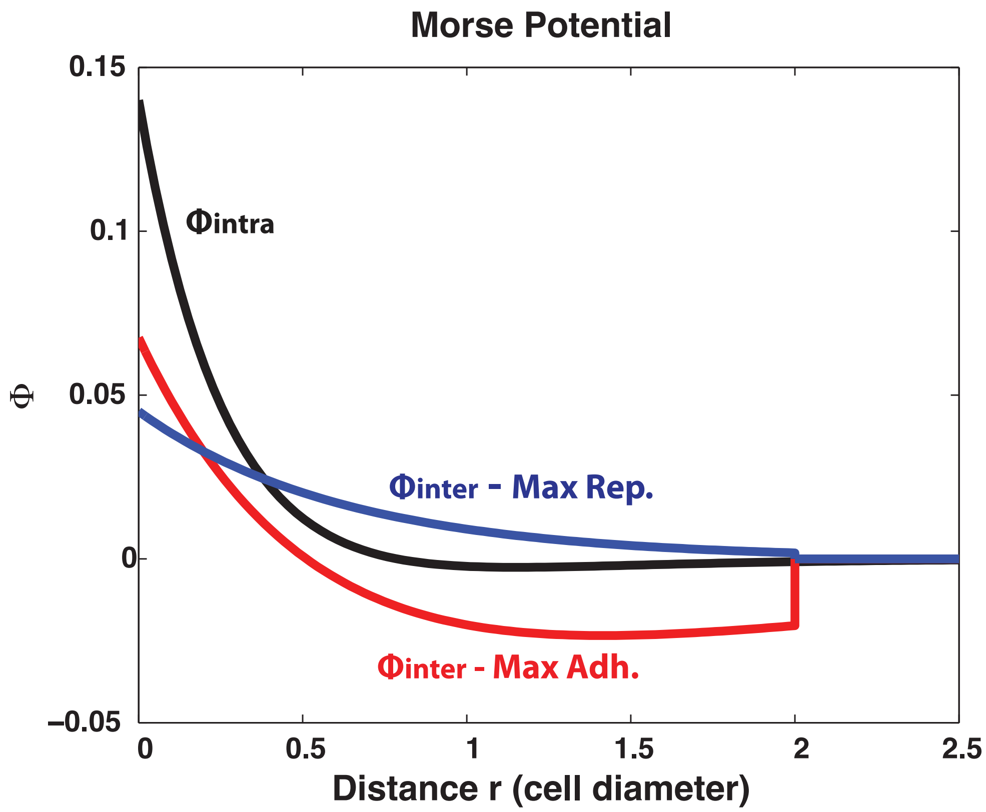

Fig C Morse potentials used in model SP. In Model SP, the intercellular forces rely continuously on the concentrations $[A]$ and $[B]$ of the cells, hence the intercellular Morse potential Φ_{inter} actually contains a continuous family of Morse potential, ranging from the maximum attraction (red line) to the maximum repulsion (blue line). All potentials are truncated beyond a length of 2 cd to ensure they represent contact based forces.

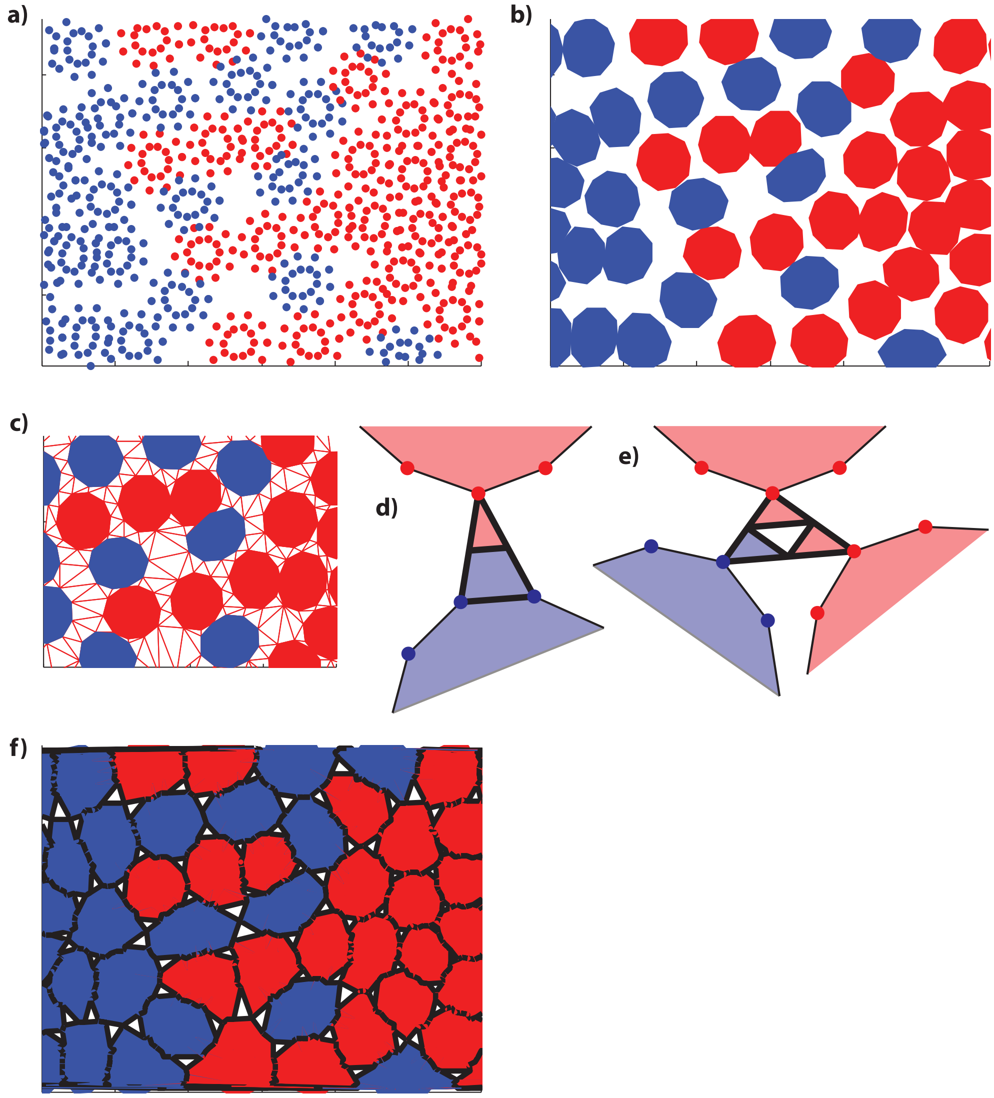

Fig D Method for drawing cell boundaries based on sub-cellular node positions. a) In our modified SCEM, a cell is represented by a collection of nodes that are structured in two concentric layers. b) Each cell is roughly defined as the region enclosed by the outer ring of nodes. c) For plotting purposes, we generate a trangulated mesh on the space between these outer cellular nodes, using the nodes as the triangle vertices. d, e) For visualization purposes, we select the mid-point along edges connecting nodes from adjacent cells and assign the subdivided regions defined by these new vertices as belonging to the cell as shown. f) Performing this globally yields a final representation of each cell that is dependent on the position of the nodes comprising that cell.

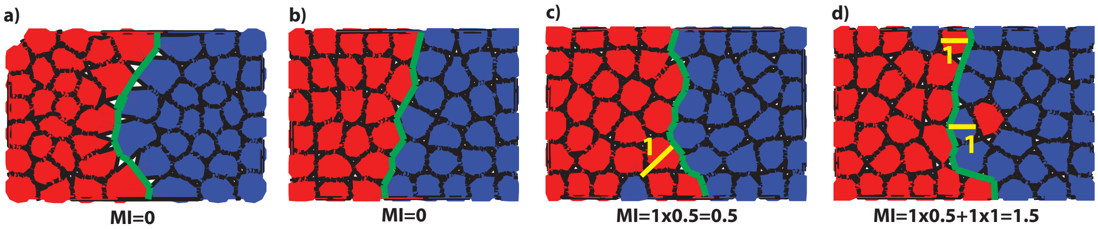

Fig E Examples of Mixture Index (MI) and Sharpness Index (SI). For MI, the green line marks the boundary in the sharp case, or the presumable one in other cases. To calculate the MI, we sum the contribution of each mislocated cell to the index. This contribution is given by the distance of that mislocated cell to the boundary (depicted by the yellow numbers and measured in cell diameters). For cells on the top or bottom boundaries, that contribution is multiplied by 0.5 to account for boundary effects.The MI and SI of each panel are a) MI=SI=0, b) MI=0, SI=0.07, c) MI=0.5, SI=0.78, d) MI=1.5, SI=1.12.

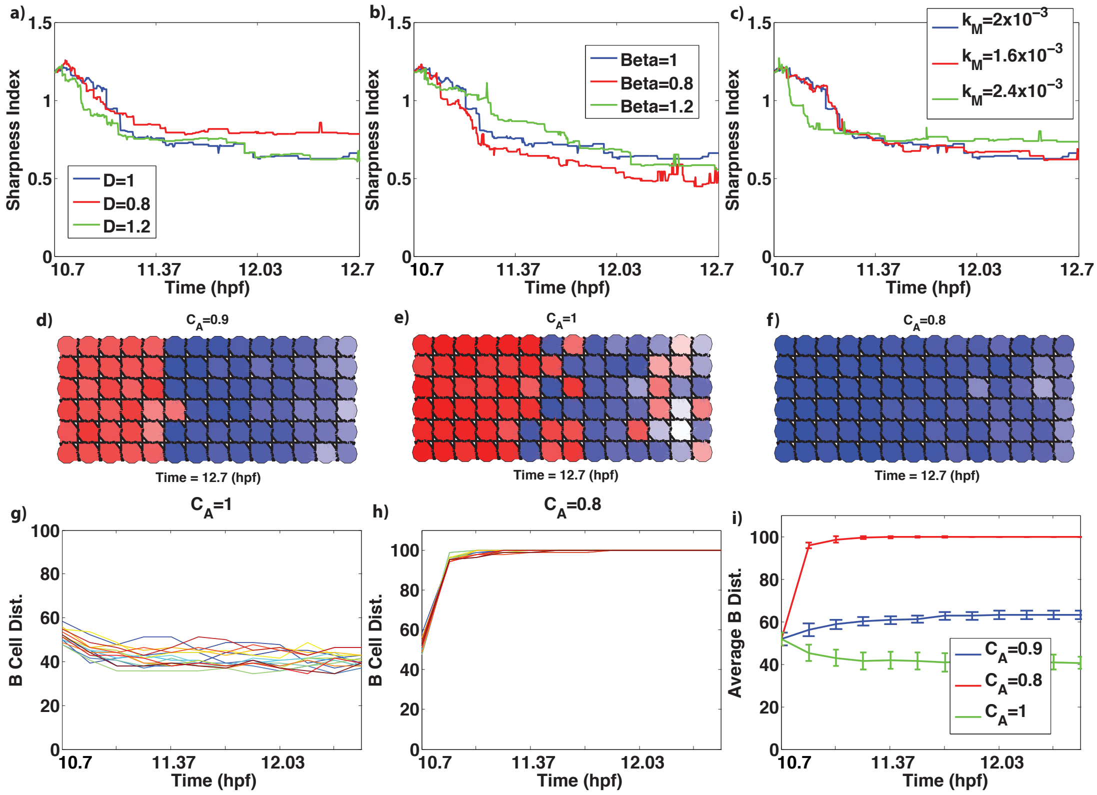

Fig F Sensitivity analysis of model P. a) The average SI changes with different values of D_M. Model P is stable to D_M: increasing or decreasing D_M by 20% will not substantially affect the system dynamics. b) The average SI changes with different values of β. Model P is stable to β: increasing or decreasing β by 20% will not substantially affect the system dynamics. c) The average SI changes with different values of k_M. d-f) The influence of C_A on sharpening. d) With $C_A = 0.9$, the transition region sharpens. e) With $C_A = 1$, sharpening fails. f) With $C_A = 0.8$, A $\rightarrow$ B transitions occur throughout the domain resulting in all cells expressing gene B at the end. g, h) Quantification of the fraction of all cells expressing the B cell fate for $C_A = 1$ and $C_A = 0.8$ respectively. Each line plots the B cell distribution from one simulation. i) The average B cell distribution of different values of C_A. For the units of the parameters, please refer to Table E.

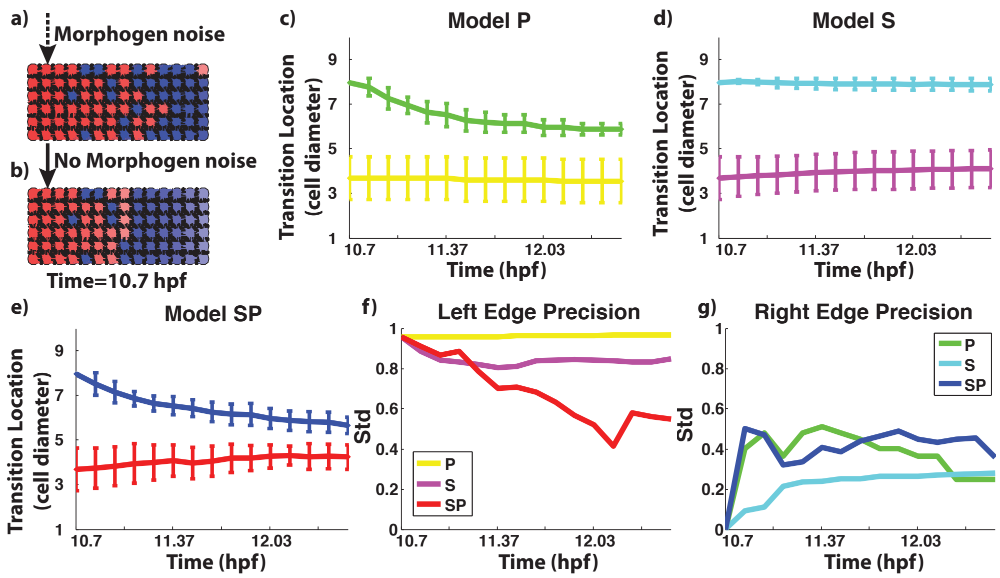

Fig G The transition region location is precise after boundary sharpening by any of the three models. a) Simulations are initialized with morphogen noise on. This leads to an initially very wide transition region. b) After the initial fate determination stage, we turn off the morphogen noise and let the system relax to reach its stable state, during which the early wide transition region is narrowed from the right side down to a narrow one typically of 3-6 cell wide. c-e) The evolution of the mean and Std of the locations of the left and right edges of the transition region for the three models (P, S and SP). f, g) The standard deviation of the left and right edge locations of the transition region for the three models.

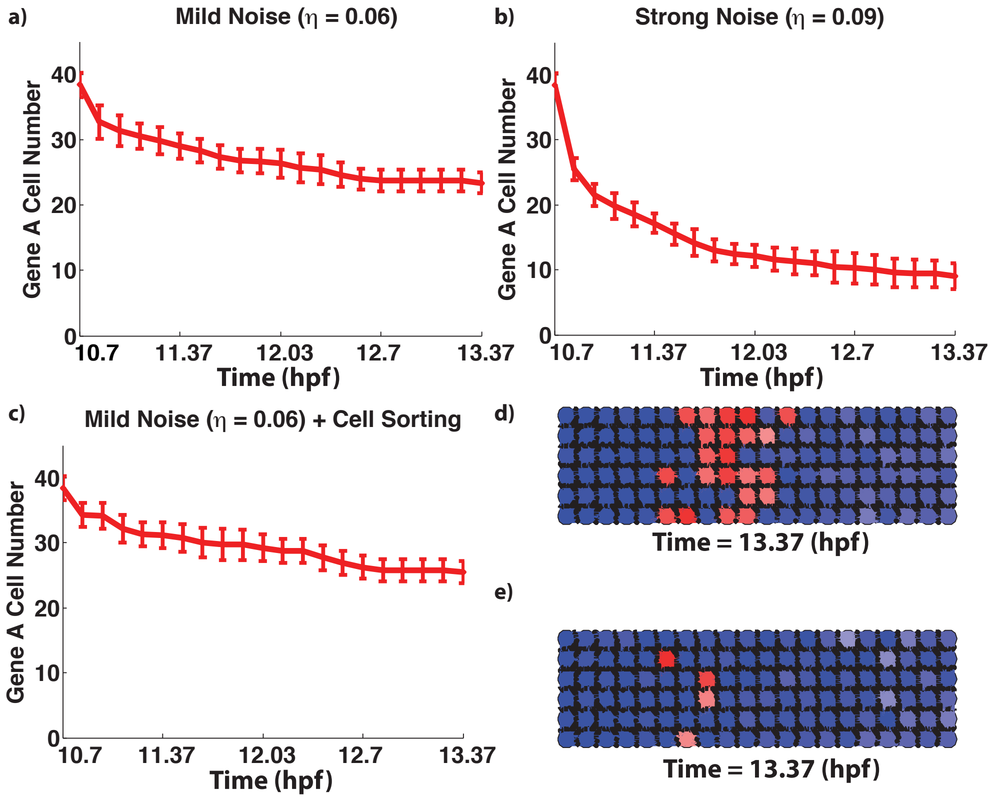

Fig H Loss of cells expression one gene caused by plasticity induced gene expression switching. In zebrafish rhombomere boundary sharpening, plasticity induced gene expression switching causes loss in cells expressing one gene, and might result in the connection of r3/4 and r4/5 transition regions. a, b) Mean and standard deviation of numbers of cells expressing gene A, in model P, with moderate and high noise levels respectively. c) Mean and standard deviation of the numbers of cells expressing gene A, in model SP, with mild noise. d) With moderate noise only and no cell sorting, r3/4 and r4/5 transition regions become connected and clear rhombomeres don't form. e) With strong noise and no cell sorting, severe loss of red cells occurs, resulting in the dominance of cells expressing gene B.

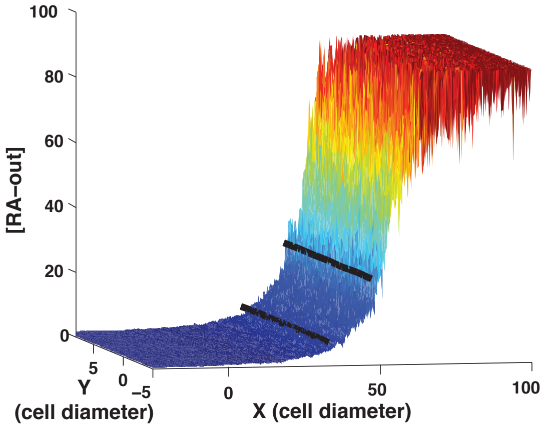

Fig I Depiction of the morphogen profile used in simulations. The section between the two black lines is shown at higher magnification in Fig 1a.

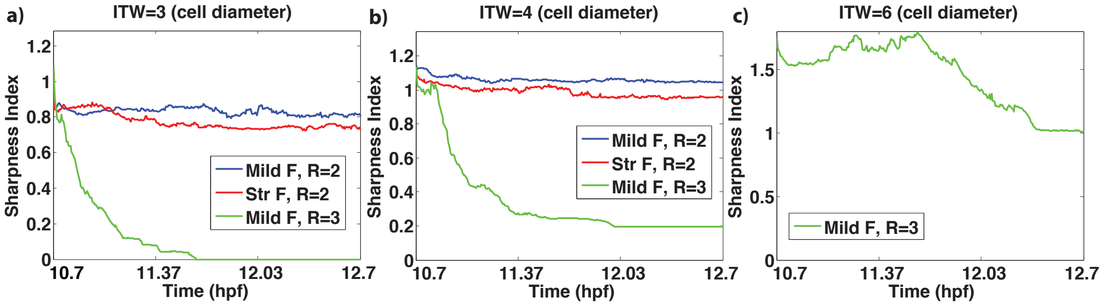

Fig J Average sharpness index (SI) over 16 simulations with mild and short-ranged (blue), strong and short-ranged (red), and mild and long-ranged (green) intercellular force, starting with different ITW. Results show that 1) increasing the strength of cell-cell interaction strengths does not improve sharpening but that 2) increasing the spatial range of those interactions (indicative of chemotactic effects) greatly improves sharpening.

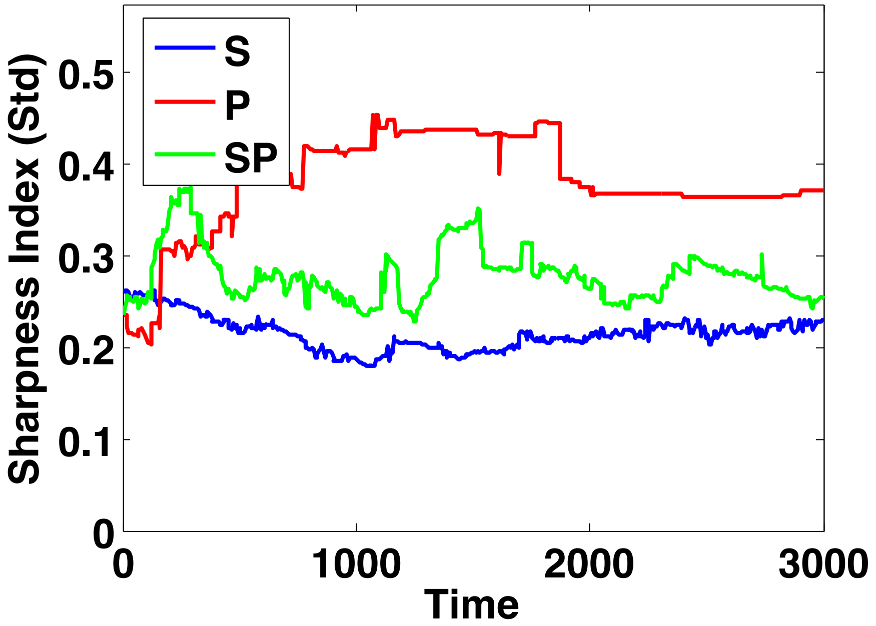

Fig K The standard deviation of SI of the model S, P and SP. The mean of SI of the models is shown in Fig 5d.

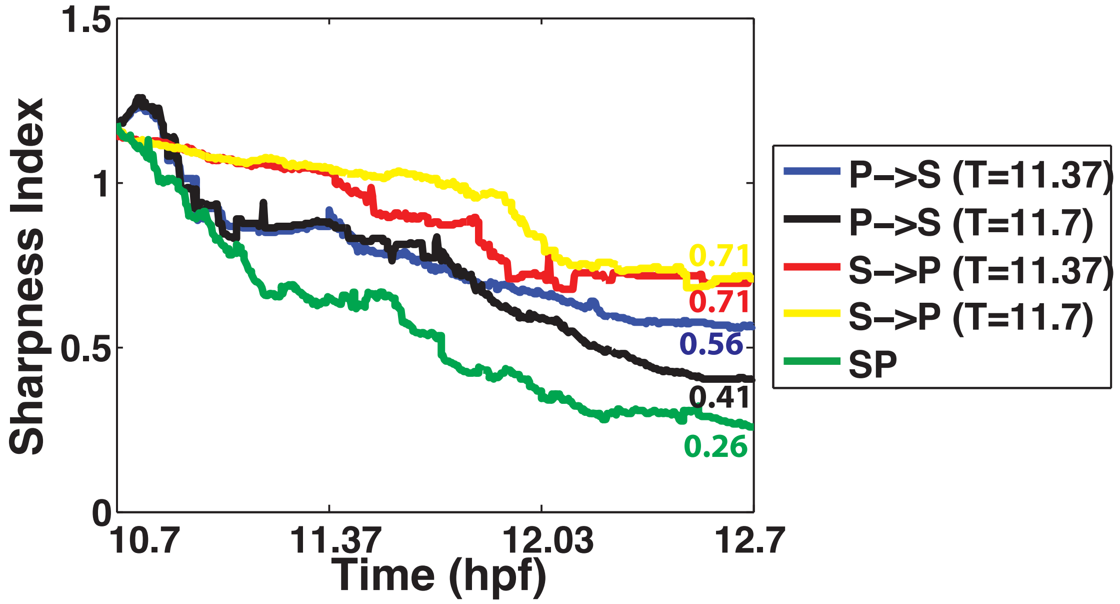

Fig L Average SI over all simulations from model P followed by S (T=11.37 hpf) (blue line), P followed by S (T=11.7 hpf) (black line), S followed by P (T=11.37 hpf) (red line), S followed by P (T=11.7 hpf) (yellow line) and SP (green line). Times here indicate the time at which the switch between different mechanisms is imposed in the simulation.

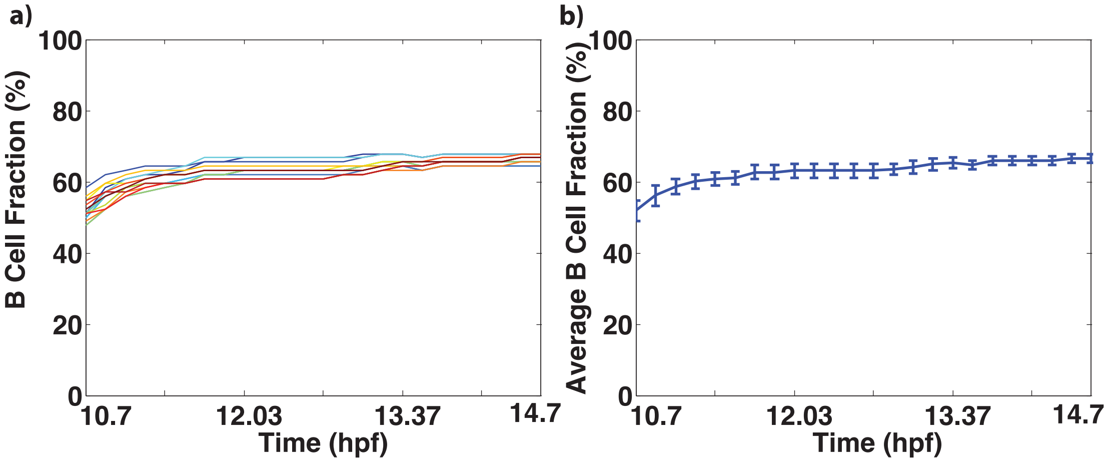

Fig M Stability analysis of the boundary formation. To assess the stability of formed boundaries over longer times, we track the fraction of cells in the domain of type B as a function of time. a) Fraction of all cells that are of type B (e.g. $\#B/(\#B+\#A)$ for individual simulations. b) Mean and standard deviation of the B cell fraction as a function of time for 16 simulations.
